# Temporal changes in zooplankton indicators highlight a bottom-up process in the Bay of Marseille (NW Mediterranean Sea)

**DOI:** 10.1371/journal.pone.0292536

**Published:** 2023-10-23

**Authors:** Théo Garcia, Daniela Bănaru, Loïc Guilloux, Véronique Cornet, Gérald Gregori, François Carlotti

**Affiliations:** Aix-Marseille Université, Université de Toulon, CNRS/INSU, IRD, Mediterranean Institute of Oceanography (MIO), Marseille, France; University of Connecticut, UNITED STATES

## Abstract

Sixteen years (2005–2020) of zooplankton monitoring in the Bay of Marseille (N-W Mediterranean Sea) are analyzed in relation to physical, meteorological, climatic and biotic data. Samples were collected every two weeks by a vertical haul (0–55 m) of a 200 μm plankton net. Different indices characterizing the mesozooplankton are compared: biomass dry weight of four size fractions between 200 and 2000 μm; abundances of the whole of the mesozooplankton and of 13 main taxonomic groups defined from plankton imagery; seasonal onset timing of each zooplankton group; and two other types of indices: the first characterized diversity based on abundance data, and the second was derived from zooplankton size spectra shape. The clearest pattern in the environmental compartment was an overall decreasing trend in nutrients, shifts in phytoplankton metrics (i.e. size structure and particulate organic matter), and changes in winter conditions (i.e. increasing temperatures, precipitation and NAO). Interannual patterns in the mesozooplankton community were: (i) a decrease of total abundance (ii) a decrease in biomass for the four size fractions, with an earlier decrease for the 1000–2000 μm size fraction (in 2008); (iii) a reduced dominance of copepods (calanoids and oithonoids) and a concomitant increase in abundance of other taxonomic groups (crustaceans, pteropods, chaetognaths, salps) which induced higher diversity; (iv) a first shift in size spectra towards smaller sizes in 2009, when the 1000–2000 μm size fraction biomass decreased, and a second shift towards larger sizes in 2013 along with increased diversity; and (iv) a later onset in the phenology for some zooplankton variables and earlier onset for salps. Concomitant changes in the phytoplankton compartment, winter environmental conditions, zooplankton community structure (in size and diversity) and zooplankton phenology marked by a shift in 2013 suggest bottom-up control of the pelagic ecosystem.

## 1-Introduction

Zooplankton are short-lived organisms, highly sensitive to environmental changes, which makes their long-term monitoring an excellent approach to provide responses to management issues [1, 2]. Previous time series analyses have shown that zooplankton shifts can be observed at various scales: at large scale through climate-related processes [3, 4], at regional scale in relation with regional meteorological phenomena, and at local scale (e.g. bays) most often in response to human pressure [5, 6]. These multiple stressors alter the physiology, behavior and phenology of organisms, and consequently population distributions [7, 8]. Nonetheless the multiple scales of forcing make it difficult to disentangle the causation.

The increase in zooplankton observations has made it possible to validate several indicators such as those based on individual taxa, species assemblages, diversity indices or size spectra, which are useful for characterizing the state of pelagic ecosystems [1]. The development of multi-indicator approaches has made it possible to better compare them, and thus to identify their complementary contributions. Therefore nowadays quantification of the roles of zooplankton in biogeochemical fluxes and trophic fluxes towards higher levels requires, in addition to information on biomass and size spectra, knowledge of taxonomic distributions and associated ecological traits.

In the Gulf of Lion (NW Mediterranean), earlier studies dedicated to zooplankton were focused on quantification of either biogeochemical flux or pelagic trophic flows, and were all carried out during oceanographic cruises over short periods (less than a month) [9–19]. Recent increased interest in the role of zooplankton as trophic resources for planktivorous fish has been prompted by the reduction in size of two planktivorous fishes (European pilchard, *Sardina pilchardus*, and European anchovy, *Engraulis encrasicolus*) in the Gulf of Lion in 2008 (NW Mediterranean) [20–23]. Various studies have led to rejection of the hypotheses of the impact of overfishing, increased predation, disease and a decline in recruitment [24]. The observation of a drastic reduction in the size of individuals [20, 24, 25] has been linked to a deficit in individual growth attributed to potential changes in plankton resources, although this has not yet been proven. To shed light on this potential planktonic change, the data from oceanographic cruises over the last few decades in the Gulf of Lion are too few and limited in time, and the closest locations of zooplankton monitoring time series in the Ligurian Sea [4, 26, 27] or the Balearic Sea [28] are far apart.

A mesozooplankton monitoring survey that has been carried out since 2005 in the Bay of Marseille (BoM) provides a zooplankton time series in the Gulf of Lion which encompasses the critical shift period. Zooplankton sampling was added to an existing monitoring programme started in 1994 including hydrological and nutrient measurements, as well as sampling for a few biotic variables (pigment, bacteria and phytoplankton). The sampling station at the center of the BoM is subject to multiple regional environmental influences such as winter deep convection phenomena from the Provencal basin off the Gulf of Lion, north-westerly winds (known as Mistral), which induce coastal upwelling events [29], intrusions from the Rhone River under certain wind conditions [30], intrusions from the northerly current from the Ligurian Sea [31], and local anthropogenic influences due to the proximity of the Marseille agglomeration (e.g. sewage plants) [32].

In this paper, we aimed to test the hypothesis of a bottom-up relationship between environmental conditions, phytoplankton, and zooplankton in the BoM. To achieve this goal we will first describe a number of zooplankton indices and examine their interannual and seasonal variations in order to identify major changes over the last two decades. We will then relate zooplankton changes to variations in other monitored abiotic and biotic parameters, and discuss how the observed changes might be the result of bottom-up forcing. Finally, we will discuss how this could affect planktivorous fish.

## 2-Material and methods

### 2.1-Environmental data

**Fig 1:**
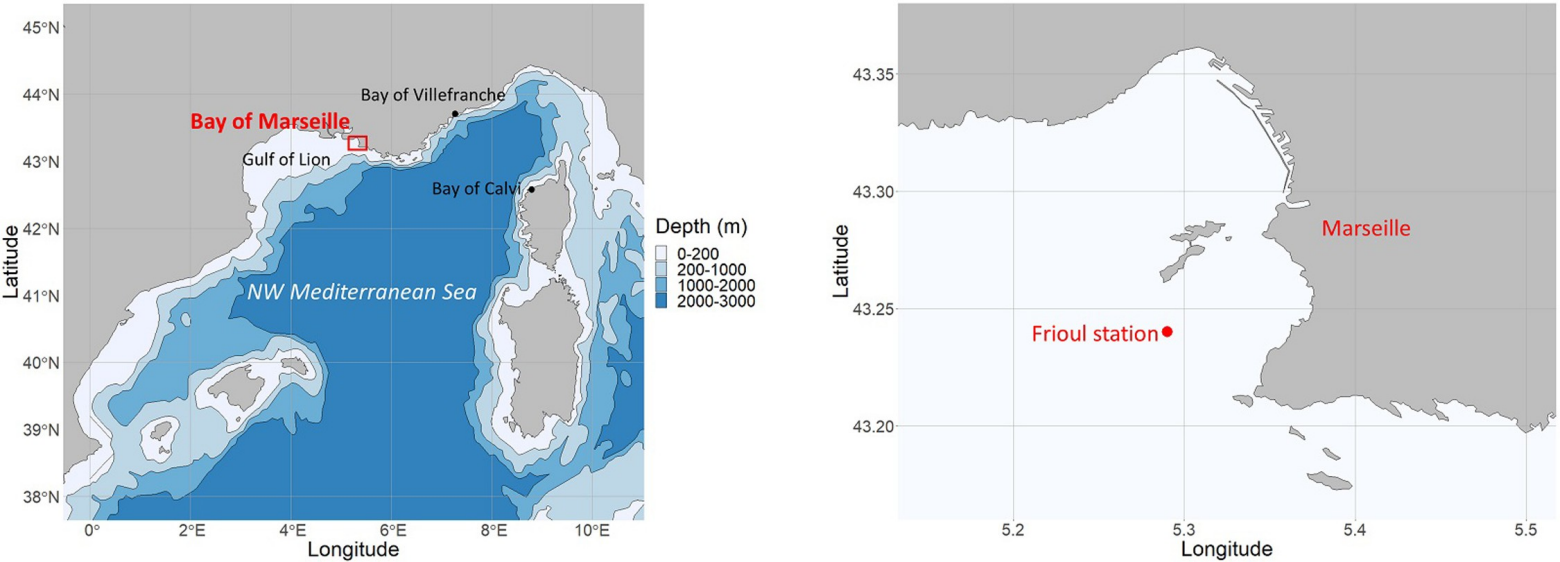
Map of the study site. In the left panel, localization of the Bay of Marseille (red) at basin (NW Mediterranean) and Gulf of Lion scale. In the right panel, localization of the Frioul sampling station in the Bay of Marseille. Color gradient refers to the bathymetry on the left panel. Maps were produced using Natural Earth and France-GeoJSON [33] open access data.

Sampling was carried out in the Bay of Marseille (N-W Mediterranean Sea) at the Frioul monitoring station (60m depth, 43.24°N; 5.29°E) of the long-term national littoral observation program SOMLIT (www.somlit.fr), in the eastern part of the Gulf of Lion (Fig 1). This monitoring survey has generated an extensive collection of physical, chemical, and biological data (see summary of the variables in Table 1). Twice a month since 1994, a vertical CTD-oxygen-fluorometer cast of the whole water column (0-55m) and Niskin bottle sampling were performed at three depths (subsurface -1 m-, bottom -55 m- and fluorescence maximum -variable). The values of each measurement obtained at the three depths were averaged. Climatic and meteorological indices completed the environmental database (Table 1).

**Table 1:** Summary of the variables used in this study.

Variable	Abbreviation	Unit	Category	Time	Source
**Wind stress (according to NW-SE axis)**	Wind stress	-	Environmental	2005–2020	Calculation from Météo France
**Precipitation**	Prec	mm	Environmental	2005–2020	Météo France
**Mixed Layer Depth**	MLD	m	Environmental	2005–2020	Calculation from SOMLIT data
**Western Mediterranean Oscillation**	WeMO	-	Environmental	2005–2020	[34]
**Northern Atlantic Oscillation**	NAO	-	Environmental	2005–2020	NOAA
**Temperature**	T	°C	Environmental	2005–2020	SOMLIT
**Salinity**	S	-	Environmental	2005–2020	SOMLIT
**Oxygen**	O	mL.L^-1^	Environmental	2005–2020	SOMLIT
**Ammonium concentration**	NH4	μmol.L^-1^	Environmental	2005–2020	SOMLIT
**Nitrate concentration**	NO3	μmol.L^-1^	Environmental	2005–2020	SOMLIT
**Nitrite concentration**	NO2	μmol.L^-1^	Environmental	2005–2020	SOMLIT
**Phosphate concentration**	PO4	μmol.L^-1^	Environmental	2005–2020	SOMLIT
**Particulate Organic Carbon**	POC	μg.L^-1^	Environmental	2005–2020	SOMLIT
**Particulate Organic Nitrogen**	PON	μg.L^-1^	Environmental	2005–2020	SOMLIT
**Suspended particulate matter**	SPM	μg.L^-1^	Environmental	2005–2020	SOMLIT
**Chlorophyll a**	CHLA	μg.L^-1^	Environmental	2005–2020	SOMLIT
**Diatoms and diatoms counts (subsurface)**	Micro	cells.L^-1^	Environmental	2010–2020	SPECIMED [35] and PHYTOBS [36]
**Ratio diatoms counts: dinoflagellates counts (subsurface)**	Ratio Diat:Dino	-	Environmental	2010–2020	SPECIMED [35] and PHYTOBS [36]
**High Nucleic Acid Bacteria counts (subsurface)**	HNA bacteria	cells.mL^-1^	Environmental	2009–2020	SOMLIT
**High Nucleic Acid Bacteria size index (subsurface)**	HNA bacteria size	-	Environmental	2009–2020	SOMLIT
**Low Nucleic Acid Bacteria counts (subsurface)**	LNA bacteria	cells.mL^-1^	Environmental	2009–2020	SOMLIT
**Low Nucleic Acid Bacteria size index (subsurface)**	LNA bacteria size	-	Environmental	2009–2020	SOMLIT
**Total Bacteria counts (subsurface)**	Tot bacteria	cells.mL^-1^	Environmental	2009–2020	SOMLIT
**Total Bacteria size index (subsurface)**	Tot bacteria size	-	Environmental	2009–2020	SOMLIT
*Cryptophycea* counts (subsurface)	Crypto	cells.mL^-1^	Environmental	2009–2020	SOMLIT
*Cryptophycea* size index (subsurface)	Crypto size	-	Environmental	2009–2020	SOMLIT
*Synechococcus* counts (subsurface)	Syne	cells.mL^-1^	Environmental	2009–2020	SOMLIT
*Synechococcus* size index (subsurface)	Syne size	-	Environmental	2009–2020	SOMLIT
*Prochlorococcus* counts (subsurface)	Pro	cells.mL^-1^	Environmental	2009–2020	SOMLIT
*Prochlorococcus* size index (subsurface)	Pro size	-	Environmental	2009–2020	SOMLIT
**Picophytoplankton counts (subsurface)**	Pico	cells.mL^-1^	Environmental	2009–2020	SOMLIT
**Picophytoplankton size index (subsurface)**	Pico size	-	Environmental	2009–2020	SOMLIT
**Nanoplankton counts (subsurface)**	Nano	cells.mL^-1^	Environmental	2009–2020	SOMLIT
**Nanoplankton size index (subsurface)**	Nano size	-	Environmental	2009–2020	SOMLIT
**Biomass for size fraction 1000–2000μm**	Biom 1000–2000	mg of dry weight.m^-3^	Zooplankton	2005–2020	This study
**Biomass for size fraction 500–1000μm**	Biom 500–1000	mg of dry weight.m^-3^	Zooplankton	2005–2020	This study
**Biomass for size fraction 300–500μm**	Biom 300–500	mg of dry weight.m^-3^	Zooplankton	2005–2020	This study
**Biomass for size fraction 200–300μm**	Biom 200–300	mg of dry weight.m^-3^	Zooplankton	2005–2020	This study
**Total Biomass 200–2000 μm**	Total Biomass	mg of dry weight.m^-3^	Zooplankton	2005–2020	This study
Zooplankton size structure (1^st^ PC)	Zoopk PC1	-	Zooplankton	2005–2020	This study
**Zooplankton size structure (2nd PC)**	Zoopk PC2	-	Zooplankton	2005–2020	This study
**Community structure index MDS 1**	MDS1	-	Zooplankton	2005–2020	This study
**Community structure index MDS 2**	MDS2	-	Zooplankton	2005–2020	This study
**Community structure index MDS 3**	MDS3	-	Zooplankton	2005–2020	This study
**Zooplankton abundances**	Abundance	individuals.m^-3^	Zooplankton	2005–2020	This study

### 2.2-Zooplankton data

In parallel with the SOMLIT monitoring survey, mesozooplankton samples have been collected since 2005 through vertical hauls using a WP2 200 μm mesh size plankton net (water column sampled: 0–55 m). The samples were fractioned in two. The first half of the cod-end content was preserved in a 4% buffered formaldehyde solution for digitalizing; the other half was maintained in cold condition for zooplankton size-fractioned dry weight (biomass) measurements. Complementary information concerning the environmental and zooplankton monitoring is given in the S1 File.

#### 2.2.1-Zooplankton size-fractioned dry weights

The aliquot of cod-end content maintained in marine water was fractioned on a sieve column in 6 size fractions: 80–200 μm, 200–300 μm, 300–500 μm, 500–1000 μm, 1000–2000 μm and >2000 μm. The material from each sieve was recovered on pre-combusted and pre-weighed Whatman GF/F filters and dried for 48h at 60°C for biomass measurement. Because the WP2 200 μm mesh size plankton net does not efficiently sample the smallest and largest size fractions, the 80–200 μm and >2000 μm fractions were not considered in our study.

#### 2.2.2-Zooplankton digitalization

The other aliquot conserved in the formaldehyde solution was used for plankton digitalization following the procedure in [37] with a Zooscan. During the image processing, a proxy of individual size was obtained by means of Equivalent Spherical Diameter (ESD, see [38]). A Convolutional Neural Network (CNN) was trained for vignette classification [39]. The CNN predicted zooplankton vignettes in the following 13 classes: appendicularians, bivalves, calanoid copepods, ergasilida copepods, harpacticoid copepods, oithonoid copepods, nauplii copepods, crustaceans (which includes holoplankton, e.g. amphipod, cladocera, and meroplankton, e.g. decapod), chaetognaths, cnidarians, fish eggs, pteropods and salps. See the S2 File for more details concerning CNN classification, training, and performance.

### 2.3-Data analysis

Data analyses were conducted on R software version 4.1.2 [40].

#### 2.3.1-Description of zooplankton community structure indices

Size spectra were constructed using images with ESDs between 200 μm (limit of particle detection by Zooscan) and 1660 μm (i.e. outliers were excluded according to the Tukey method [41]). Following the method described by Nerini and Ghattas [42], the shape of the zooplankton size spectra was studied by means of Functional Principal Component Analysis (FPCA). The FPCA is a statistical tool that ordinates sample units according to the characteristics (i.e. the shape) of their functional entity (i.e. here a curve/size spectrum). This methodology was applied as follows: (i) zooplankton size spectrum densities for all samples were estimated using Kernel density estimator; (ii) the density functions were converted into a continuous functional object, a curve, using 250 B-splines functions; (iii) the curves obtained from all samples were ordinated by means of a FPCA [43]. We applied the FPCA on the coefficients of the B-spline expansion model as explained by Pauthenet et al. in [44]. These new variables which concentrate the variance of the system are the principal components (PCs) and represent the most significant modes of the data variation.

The sample scores on the first two PCs were used as time series to describe the variations of the size spectrum shapes and by extension the structure of the zooplankton community size.

In addition, multidimensional scaling (MDS) was applied on the species abundance table to disentangle the diversity structure with Manhattan distance. The sample scores of the three MDS axes were used as time series to describe the variations of the diversity structure.

The R packages ’fda’ [43] and ’vegan’ [45] were respectively used to perform the FPCA and MDS analysis.

#### 2.3.2-Exploring interannual changes in zooplankton data

Time series data were averaged on a monthly basis and missing values were imputed using the Multiple Imputation with Principal Component Analysis (MIPCA) procedure using the R package ’MissMDA’ [46]. Trends in monthly time series were highlighted using Local Polynomial Regressions (LOESS, using 75% of the neighborhood data). Breakpoint detection procedure (‘strucchange’ package in R [47]) was applied to highlight the structural change on every single time-series. Until three breakpoints were investigated, the optimum number of breakpoints was defined according to the Bayesian Information Criterion (BIC).

#### 2.3.3-Zooplankton seasonality

Level plots were used to describe seasonal patterns in the various zooplankton series: biomass, abundance (see 2.2- Zooplankton data) and structure descriptors (see 3.1- Community structure indices). In order to obtain a smoothed picture of the level plot, LOESS functions were applied over 30 equally spaced points on the year/month grids. Seasonal patterns were assessed by fitting sinusoidal models. The seasonal pattern was considered significant when the sinusoidal effect was significant (p-value<0.05). The seasonal onset timing of each zooplankton group was defined as the date when 20% of the yearly cumulative value was reached [8, 48].

#### 2.3.4-Investigating relationships between environmental and zooplankton time series

Dynamic Factor Analysis (DFA, [49]) is a statistical method that aims to find common trends among observational data. In our case DFA was applied in order to find common trends between environmental and zooplankton data. DFA belongs to the family of the state-space models and allows determination of M hidden common trend(s) among N standardized (to reduce the impact of the variable scale on the analysis) data time series, when M<<N. DFA models relate observational time series (*y*) to the sum of the M hidden trends linear combination and noise. This can be represented by the following Eq (1):

$$y_{t}=\Gamma \alpha_{t}+\varepsilon_{t};\;where\;\varepsilon_{t}∼N(0,R)$$ (1)

Γ is a matrix of dimensions N x M and contains the factor loadings of the N factors for M detected hidden trends, α_t_ is a vector of length M containing the M common trends at time t. ε_t_ is the noise component which follows a normal distribution of mean 0 and variance-covariance matrix R.

DFA models require user specification for certain parameters:

- Firstly, different assumptions on the noise, R, variance-covariance matrix among factors can be made:
  - same variance and no covariance (‘diagonal and equal’)
  - different variances and no covariances (‘diagonal and unequal’)
  - same variances and same covariances (‘equalvarcov’)
  - different variances and covariances (‘unconstrained’)
- Secondly, the number of M hidden trends must be specified as low as possible to facilitate interpretation (M<<N).

Correction of Akaike Information Criterion (AICc) was applied to select the most likely data model.

Multiple DFA models were computed on:

‐ all environmental data for the 2005–2020 period. From one to three common trends were investigated among the 34 environmental times series with the four different assumptions on the R matrix. A total of 12 models were computed on environmental time series.
‐ zooplankton data for the 2005–2020 period. From one to three common trends were investigated among 11 zooplankton times series with the four different assumptions on the R matrix. A total of 12 models were computed on biological data.
‐ environmental winter conditions (average values between January and March) and dates of zooplankton seasonal onset. From one to three common trends were investigated among the 34 environmental and 19 zooplankton time series with the four different assumptions on the R matrix. A total of 12 models were computed.

As DFA aimed at finding common trends among short time series, in (1) and (2), time series were averaged at a biannual time step. This allowed us to reduce the computing time for the models. The series were de-seasonalized (i.e. when seasonal patterns were detected, by means of partial autocorrelation functions) to focus on interannual variations. DFA can be performed with missing data. For models 1 and 3 some time series were shorter (i.e. micro-, pico-, nano- plankton data started in 2009); the results of the models computed for those variables were therefore not discussed before 2009.

Dynamic factor analysis models were computed with the MARSS package [50].

## 3-Results

### 3.1-Community structure indices

**Fig 2:**
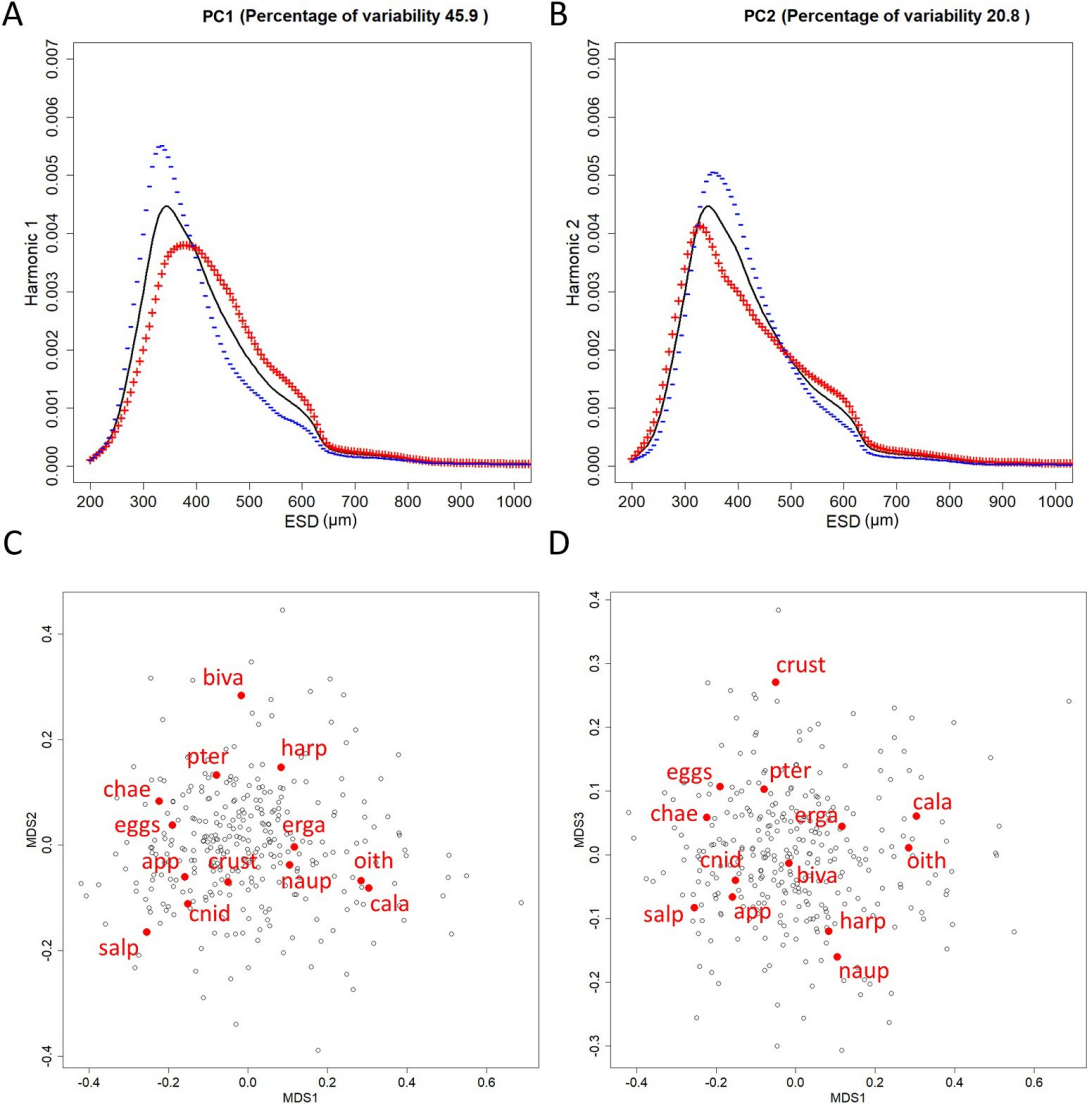
Results of the FPCA (A & B) and MDS (C & D) analysis to derive community structure indices. Influence of factorial axis (A for first PC and B for second PC) on the shape of the functional object (size spectra curve). Black line is the mean functional curve of the FPCA. Red curve (+ symbol) and blue curve (- symbol) represent respectively the positive and negative influence of the factorial axis on the size spectra shape. Limits of the x-axis of A & B graphs are set to 200 to 1000 μm, as the main variations of the influence of the factorial axis on the functional object are observable before 1000 μm ESD. Representation of the ordination of the MDS (C: first vs second axis, D: first vs third axis). The black dots correspond to the projections on the 2D space of the samples. The red labels correspond to the centroids position of the *taxa*. *Taxa* abbreviations correspond to the following groups: app: appendicularians; biva: bivalves; cala: calanoids copepods; chae: chaetognaths; cnid: cnidarians; crust: crustaceans (without copepods); eggs: fish eggs; erga: ergasilida copepods; harp: harpacticoids copepods; naup: copepods nauplii; oith: oithonids copepods; pter: pteropods; salp: salp.

The two first PCs of the FPCA performed on the size spectra curves explained 66.7% of the data variability (Fig 2A and 2B). In each figure, positive and negative influences of the factorial axes on the size spectra shape are displayed. The variation modes of the size spectra shape on the first axis explained 45.9% of the data variability. Positive values on the first factorial axis (Fig 2A, red curve) were associated with a displacement of size spectra mode towards higher ESD. Negative values on first factorial axis (Fig 2A, blue curve) were associated with a displacement of size spectra mode toward lower values. The second axis of the FPCA explained 20.8% of the data variability. Positive values on the second PC (Fig 2B, red curve) were associated with the displacement of the size spectra peak toward lower ESD and the increase of the proportion of larger zooplankton organisms (>500 μm ESD). Negative values on the second PC (Fig 2B, blue curve) were associated with the displacement of the size spectra peak toward higher ESD and the decrease of the proportion of larger zooplankton organisms (>500 μm ESD). The MDS (Fig 2C and 2D) computed on the abundances of the thirteen taxa best recognized by the CNN model summarized the structure in species of the zooplankton community (stress <0.2). The ordination method separated in the first axis the centroid of two copepod taxa (calanoids and oithonoids, positive values) to chaetognaths and salps (negative values). The second axis (Fig 2C) separated bivalve, pteropods and harpacticoid centroids (positive values) from all other species centroids. The last axis (Fig 2D) discriminated crustacean centroid (positive values) from copepods nauplii centroid (negative values).

### 3.2-Zooplankton interannual variations and relation with the environmental trend

**Fig 3:**
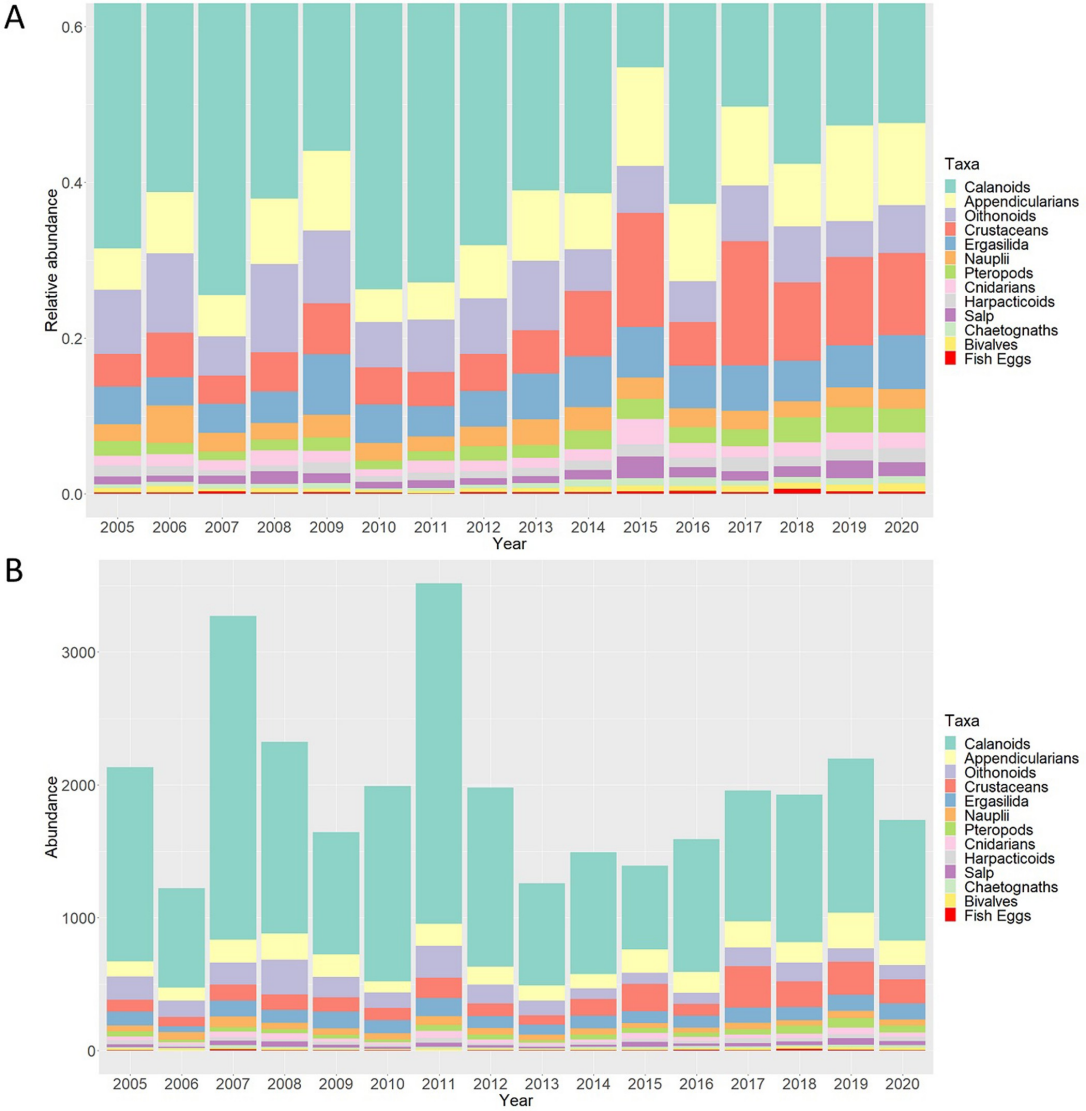
Year-to-year variations in the A) relative and B) absolute abundance of the 13 best recognized zooplankton taxa. Note that the graph is zoomed on the y values below 0.6 to better see the species with low relative abundance. Abundances in the bottom panel are expressed as number of individuals.m^-3^. The taxa are sorted by importance over the whole zooplankton time series.

**Fig 4:**
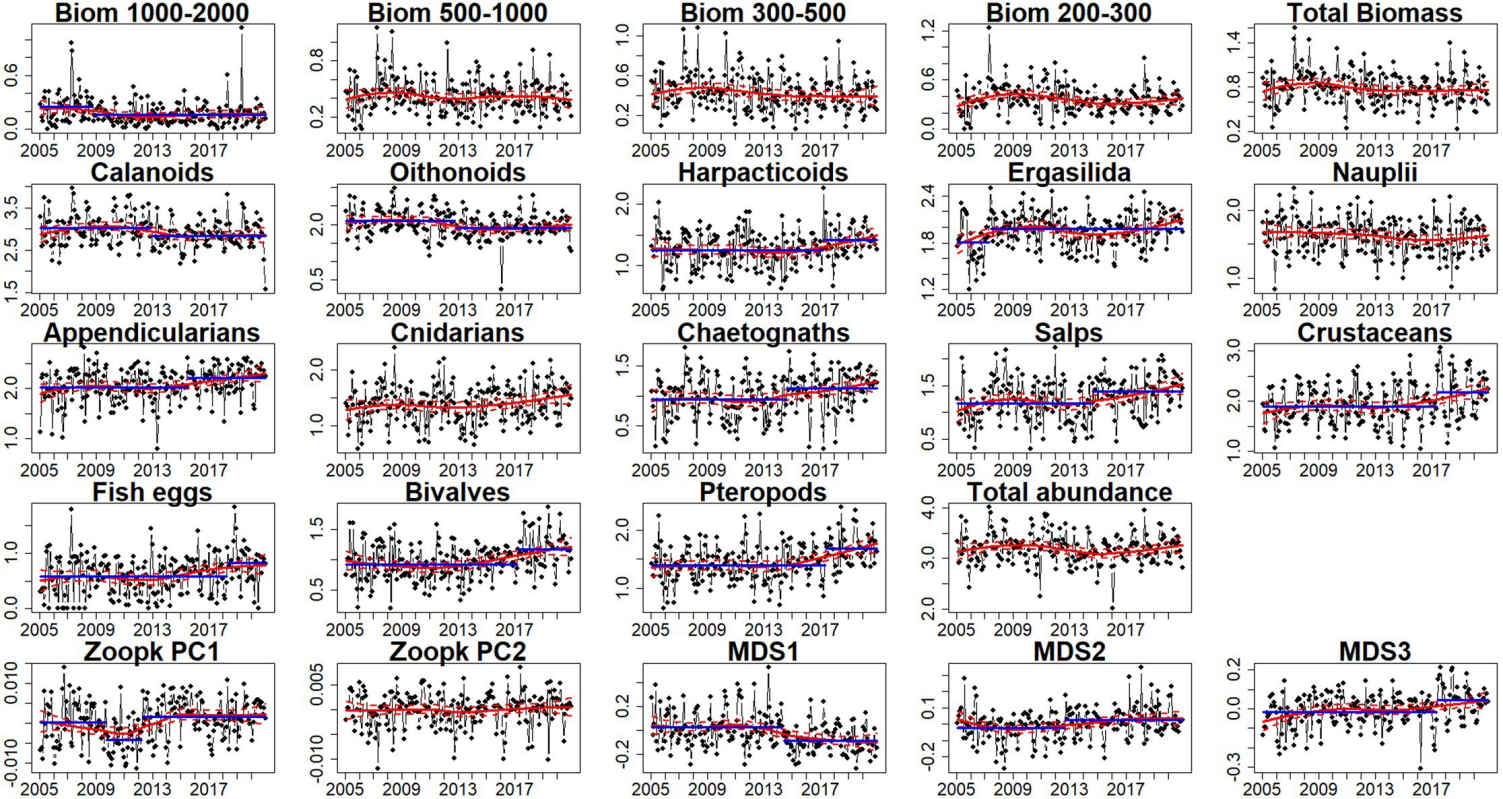
Representations of the monthly interannual times series of the zooplankton variables (black dots). The red continuous (and dashed) lines correspond to the smoothing (and its 95% confidence interval) by means of local regressions to describe the trends. The blue lines correspond to the mean values of the series before and after a breakpoint date. For series without blue lines, no breakpoints were detected. Series of size-fractions biomasses, in mg.m^-3^, (Biom 1000–2000, Biom 500–1000, Biom 300–500, Biom 200–300 μm), *taxa* abundances and total zooplankton abundance, in individual.m^-3^, are log transformed (log10(x+1)).

The zooplankton community in the Bay of Marseille is dominated by calanoids (Fig 3A) which represent more than 50% of the individuals sampled. The second most represented taxa were appendicularians, oithonoids and crustaceans which represent with calanoids, on average, more than 80% of the zooplankton community identified in this study. Obvious changes in relative abundance of some species occurred over the observation period. The dominance of calanoids seemed to have declined from more than 60% before 2012 to 50% after 2017, in contrast to the relative abundance of crustaceans which almost doubled from 5 to 10% during the same period. The changes in the relative abundances were mainly attributed to the diminution of copepod calanoid and oithonoid absolute abundances (Fig 3B). More details concerning the patterns of variation in the zooplankton community structure in species were observable on the interannual series depicted at monthly scale (Fig 4). The abundances of bivalves, chaetognaths, harpacticoids, crustaceans, pteropods, salps, appendicularians, fish eggs, and cnidarians seemed to have increased during the study and breakpoints were detected for all these taxa (except for cnidarians) in the last five years of the time series. The series of the two main copepod taxa (calanoids and oithonoids), copepod nauplii and total zooplankton abundance showed a diminution, and a breakpoint was detected for calanoids and oithonoids series at the end of 2012 (in November and September). Abundance estimations of ergasilida presented a breakpoint in April 2007, resulting in an increase in abundance. Interannual variations of the sample scores on the first MDS axis diminished (with a breakpoint detected in April 2014); this observation was based mainly on the diminution of the copepod dominance while abundances of other taxonomic groups such as chaetognaths and salps increased. The second axis highlighted the increase in abundances of bivalves, harpacticoids and pteropods (breakpoint in October 2012). The third axis represented the diminution of nauplii and increase of crustaceans abundance with the breakpoint in April 2017. The smoothing of the first axis of the FPCA revealed that after a displacement toward lower ESD values (between October 2009 ‐ March 2012), the size spectra shifted toward higher ESD after March 2012. No outstanding interannual variations of the second PC were observable and no interannual structural change of the series was detected. The biomass of the 1000–2000 μm size-fraction presented a breakpoint associated with a decrease in September 2008. The three other zooplankton biomass size fractions decreased later, but no breakpoints were detected.

**Fig 5:**
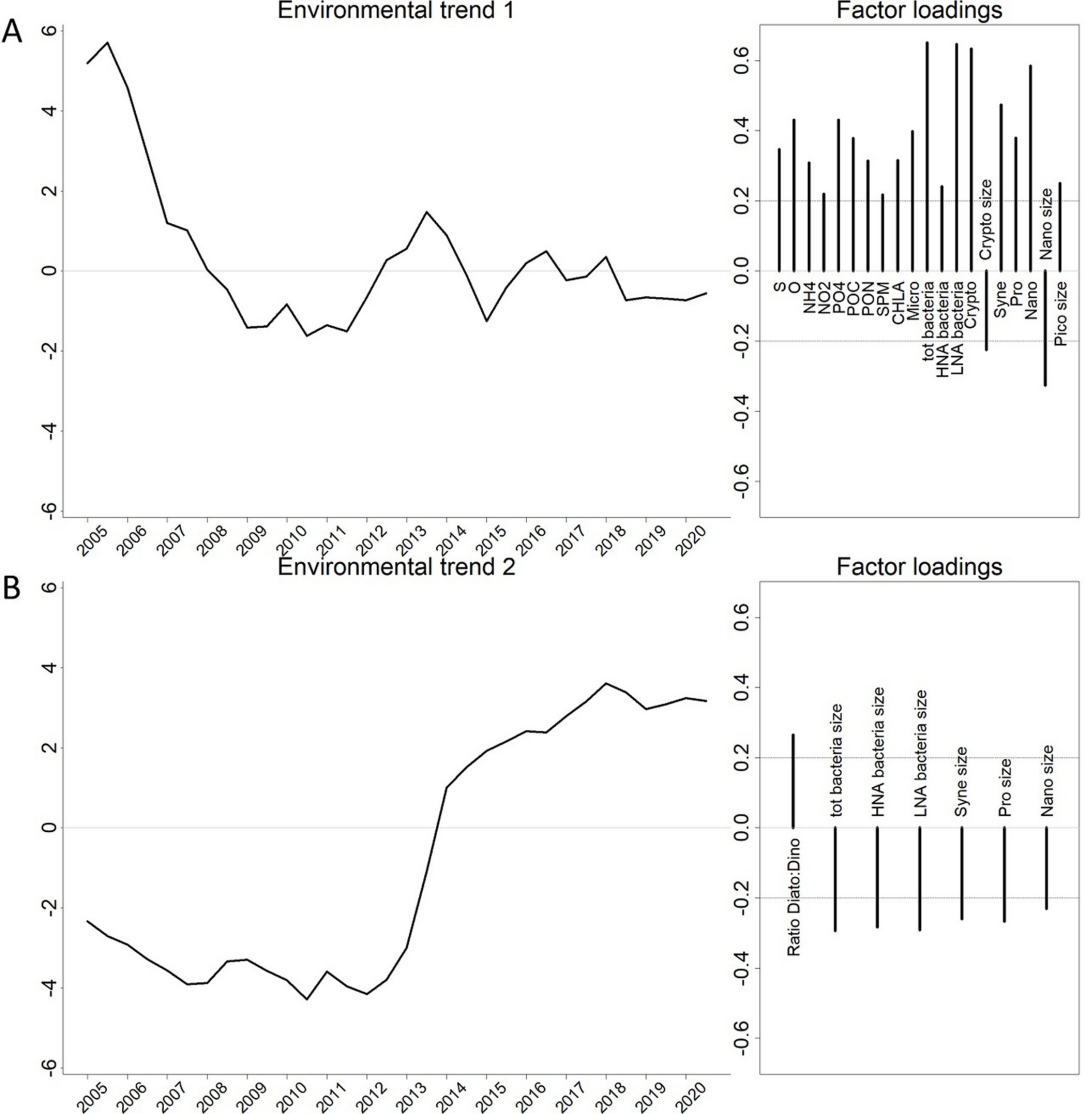
The two trends depicted by the best DFA model (diagonal and equal R matrix and two trends) on environmental data (2005–2020) and factor weights associated with each trend. Variables with positive weights are associated positively and depicted similar trends to those shown in the left panels. Inversely, variables with negative weights displayed inverse trends. Note that only the most significant factors (i.e. with weight higher than 0.2 in absolute values) are represented. See Table 1 for abbreviations of factors.

On the 12 models performed on the environmental data for the 2005–2020 period that have converged, the DFA model with a diagonal and equal R matrix and displaying two trends was considered as the best data model (see S3 File). The first trend (Fig 5A) was (a) positively characterized by variations in S, concentrations of O_2_, NH_4_, NO_2_, PO_4_, POC, PON; and abundances of bacteria (tot, HNA and LNA), *Crypthophycea*, *Synechoccocus*, *Prochlorococcus*, pico-, nanoeukaryotes and microphytoplankton, and (b) negatively characterized by variations in the size index of *Crypthophycea* and nanoeukaryotes. This trend decreased from 2005 to 2009, increased suddenly between 2011–2014 and decreased again slowly until 2021. The second trend (Fig 5B) of this model was characterized by a sudden increase between 2013 and 2014. Diatoms: dinoflagellates ratio was positively weighted while size indices of bacteria, *Synechococcus*, *Prochlorococcus* and nanoeukaryotes were negatively weighted. The fit of the data model to the environmental time series is represented in S3 File.

**Fig 6:**
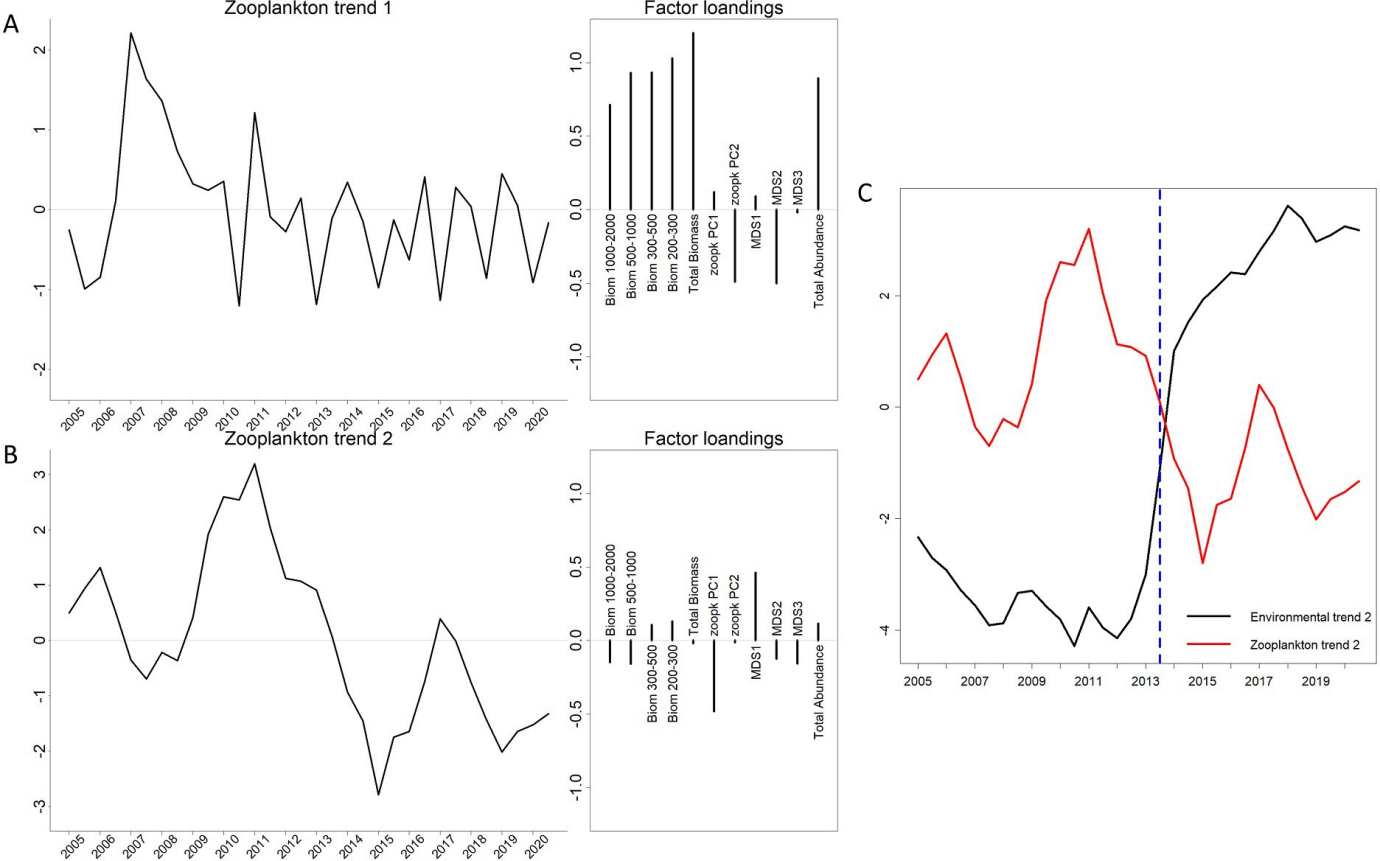
The two trends depicted by the best DFA model (diagonal and unequal R matrix) on zooplankton data (biomass, size and diversity structure indices and total zooplankton abundance series) between 2005–2020 and factor weights associated with each trend. Variables with positive weights are associated positively and depicted similar trends to those shown in the left panels. Inversely, variables with negative weights displayed inverse trends. A) and B) represent respectively the first and second zooplankton trends with their factor weights. C) trend 2 of zooplankton (in red) and trend 2 of environment (in black, see Fig 5B) are represented together. Vertical dotted blue line corresponds to the common breakpoint detected between both trends.

All 12 models performed on the zooplankton data over the 2005–2020 period converged (see S3 File). The model with two trends and a diagonal and unequal-type R matrix was the ’best’ data model deployed here. The first trend of this model peaked in 2007 and then decreased (Fig 6A). This trend displayed mainly positive variations of biomass series for every size fraction and total zooplankton abundance, and negative variations of the second size structure indicator. The second trend (Fig 6B) was characterized by a shift (decrease) between 2013 and 2014. This trend allowed depiction of positive changes in the diversity community structure index (first MDS axis) and negative changes in the size community structure index (first FPCA axis). In Fig 6C, trend 2 of zooplankton (in red) and trend 2 of environment (in black, see Fig 5B) were represented. Both trends presented a common breakpoint in mid-2013. This common breakpoint highlighted concomitant shifts in phytoplankton metrics (i.e. size, and diatoms: dinoflagellates ratio) and zooplankton community structure (i.e. size structure and diversity). The fit of the data model to the zooplankton time series is represented in S3 File.

### 3.3-Zooplankton seasonality and relationship between spring-summer production and winter environment

**Fig 7:**
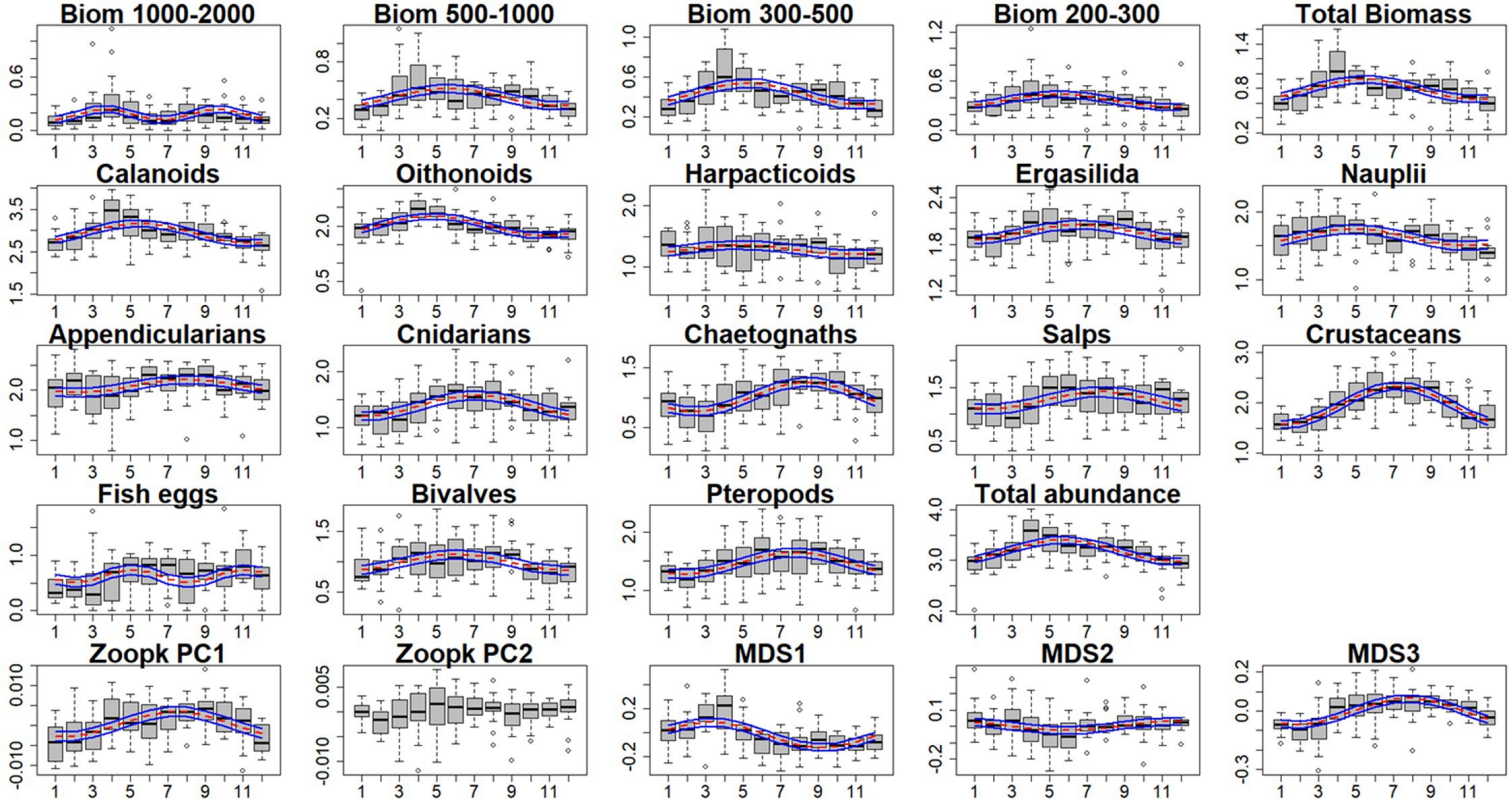
Boxplot of the seasonal (monthly) pattern of the zooplankton time series. When a model (with one or two cycles) was significant, the average prediction was represented by means of the red dashed line. The 95% confidence interval is represented by the blue lines. Series of size-fractions biomasses, in mg.m^-3^, (Biom 1000–2000, Biom 500–1000, Biom 300–500, Biom 200–300 μm, Total Biomass), *taxa* abundances and total zooplankton abundance, in individual.m^-3^, are log transformed (log10(x+1)).

Most of the zooplankton series presented a clear seasonal pattern (Fig 7) but with different shapes according to groups and size fractions. Seasonality assessments were significant (p-values<0.05) for every variable except for the second axis of FPCA (see S4 File). The total zooplankton abundance and biomass, the four-biomass size- fractions between 200 and 2000 μm, and the sample scores on the first axis of the MDS followed a similar seasonal pattern to calanoids, oithonoids, ergasilida, bivalves, copepod nauplii and harpacticoids with their highest abundance values in spring (April-May). The remaining zooplankton groups (appendicularians, chaetognaths, cnidarians, crustaceans, pteropods, salps, fish eggs) and the two remaining MDS axes seemed to reach their highest values during summer.

**Fig 8:**
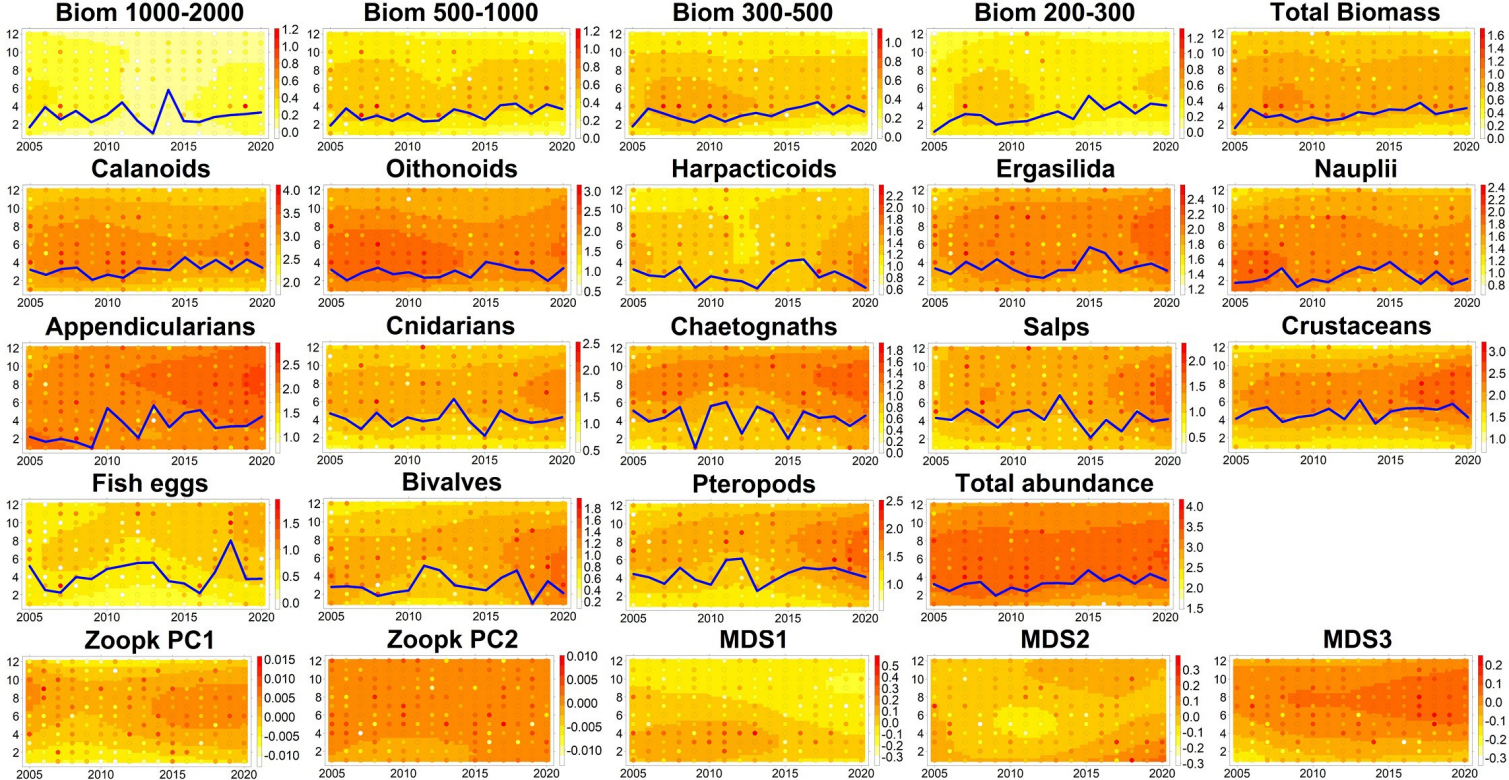
Level plots of the monthly interannual times series of the zooplankton variables. Circle colors correspond to the month value and the background color corresponds to smoothed values with LOESS function over 30 equi-spaced points. Series of biomass size-fractions, in mg of dry weight.m^-3^, (Biom 1000–2000, Biom 500–1000, Biom 300–500, Biom 200–300 μm, Total Biomass), *taxa* abundances and total zooplankton abundance, in individuals.m^-3^, are log transformed (log10(x+1)). Year-to-year variations of the date of seasonal onset, considered as 20% of annually cumulative value for biomass and abundance time series, are represented by the blue lines.

Fig 8 displays information regarding the patterns of change in the timing of the seasonal onset the different taxonomic groups. A delay in the biomass size fraction between 200 and 1000 μm, total biomass and abundance, calanoids, ergasilida and appendicularians was observable while salps tended to start their summer peak earlier. The other zooplankton groups did not present such remarkable patterns.

**Fig 9:**
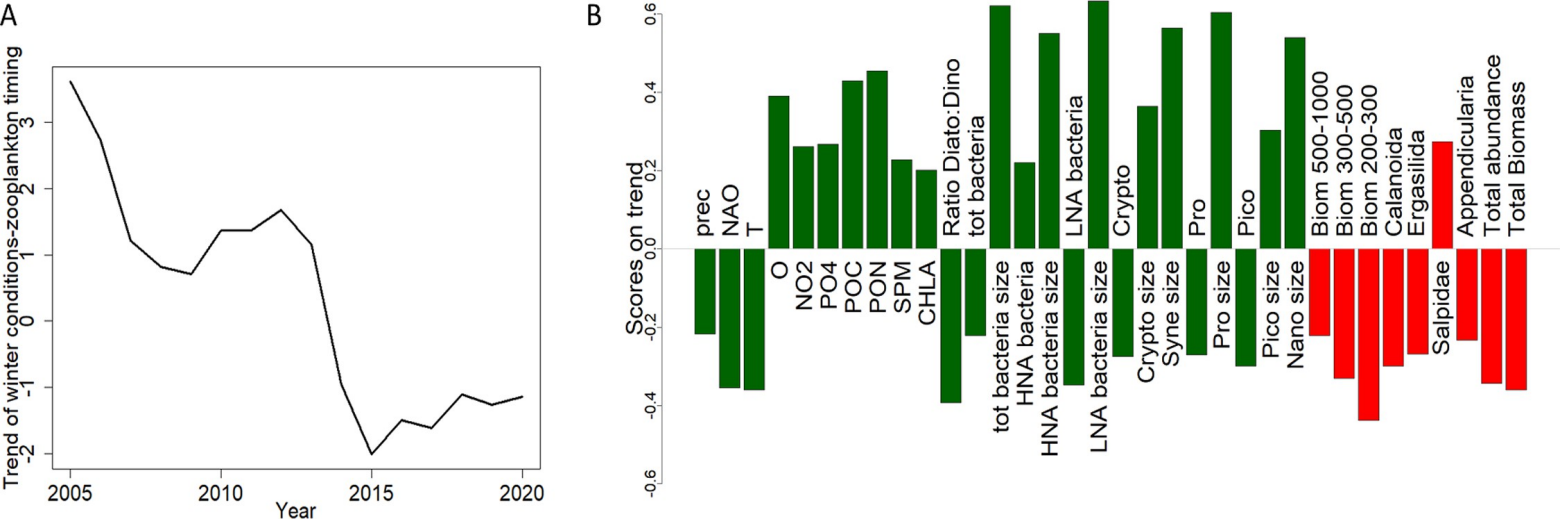
Results of the best DFA model (diagonal and equal R matrix, one trend) performed on environmental winter conditions and dates of zooplankton seasonal onset time series (2005–2020). A) The trend depicted by the DFA model. B) Baplot of the factors with the higher weights (higher than 0.2 in absolute values) are represented. Green and red bars represent respectively environmental and zooplankton series.

Fig 9 presents the results of the DFA performed under winter (January to March) environmental conditions and the timing of zooplankton seasonal onset. This highlights a common decreasing trend between the zooplankton phenology and the environmental compartment. This trend was characterized with two plateaus between, 2009–2013 and 2015–2020. During the studied period winter precipitations, NAO values, temperature, Diatoms:Dinoflagellate ratio, and abundances of bacteria, LNA bacteria, *Cryptophyceas*, *Prochloroccus* and Picophytoplankton increased. *A contrario*, winter concentrations of oxygen, nitrite, phosphate, particulate organic matter (POM), SPM and CHLA, abundances of HNA bacteria and sizes of bacteria (total, HNA and LNA), *Cryptophyceas*, *Synechococcus*, *Prochlorococcus*, Picophytoplankton and Nanoplankton decreased. To a lesser extent, MLD and microphytoplankton abundance were positively associated with this trend (but not represented in the figure because of their low weight 0.19<0.20, see S3 File). The interannual variations of winter environmental conditions were associated with on one hand, a later seasonal onset of biomass size fractions between 200 and 1000 μm, total biomass, and abundances of calanoids, ergasilida and appendicularians and, on the other hand, an earlier seasonal onset of salps.

## 4-Discussion

### 4.1-Seasonal and interannual variations of multiple zooplankton indicators

The seasonal patterns of the main zooplankton groups in the Bay of Marseille during the time series are consistent with zooplankton successions observed in Mediterranean coastal areas [5, 27, 51–54], with a zooplankton community dominated by copepods in response to the spring phytoplankton blooms and a second late summer ‐ fall peak of abundance. In the Gulf of Lion, phytoplankton-zooplankton peaks are associated for both periods with strong grazing activity [14]. The second peak is associated with higher diversity indices (as shown in Figs 7 and 8) linked to a relative diminution of the copepod contribution (mainly calanoids and oithonoids) and the appearance of other taxonomic groups (either herbivorous taxa feeding on small size phytoplankton, such as salps and pteropods, or carnivorous taxa, such as chaetognaths, crustaceans and cnidarians), a general pattern already described in the NW Mediterranean Sea [11]. In addition, this result showed a reciprocal relationship between total abundance/biomass (mostly driven by copepods) and diversity at the seasonal and interannual scales. This relationship was already observed in the Mediterranean Sea and in the global ocean [55, 56].

Our results highlight a clear shift in the zooplankton community structure (size and taxonomic assemblage) in 2013. The community structure index, MDS 1 axis, clearly represented the variations in dominance of copepods calanoids and oithonoids compared to other taxa. Since the MDS 1 index aims at summarizing dynamics of multiple taxa, it is obvious that some breakpoints are observed later or earlier when investigating interannual variations of univariate time series. Note that due to the statistical method, the dates of the different breakpoints are associated with confidence intervals, which may span multiple years depending on the series. Nonetheless the MDS 1 index summarizes the most significant patterns of zooplankton community dynamic at the seasonal and interannual scale (Figs 3 and 4 and 7 and 8). Our results are consistent with the zooplankton dynamics of the Bay of Calvi [27] and the Bay of Villefranche-sur-Mer [54] during the same decade. The main changes in the zooplankton community in the Bay of Marseille observed in 2013 (see Figs 4 and 6) appear to have occurred earlier than similar variations in the whole mesozooplankton community observed after 2015 at Villefranche-sur-Mer [54], where crustaceans (including copepods in their study) showed a diminution of abundance and a lower size spectrum slope (mainly due to the loss of small copepods). In their analysis of multiple time series in the Mediterranean Sea, Berline et al. [5] did not find any common zooplankton interannual patterns at basin scale. Nevertheless, our results and those at Villefranche-sur-mer and Calvi suggest that the coastal regions of the Ligurian Sea might be similarly impacted by the environmental changes and by their connections through the Ligurian current [57]. As the re-analysis of time series with recent data proved that some environmental-biological processes relationships may break down [58], reanalyses of zooplankton time series in the Mediterranean Sea with longer time series are required to assess some large-scale processes.

Our results showed different interannual patterns among the size-fraction biomass dynamics (see Fig 4). The sudden decline in 2008 for the largest size-fraction (1000–2000 μm) was not simultaneously observed for the three other fractions (between 200 and 1000 μm) which decreased between 2 and 4 years later. No major change in the community diversity was observed in 2008, while the diminution of the larger size fraction biomass was accompanied by a shift in the size structure of the zooplankton community. Previous studies in the Bay of Marseille have shown that the taxonomic assemblages are not the same in the four size fractions [59, 60], impacting differently the biomass dynamics of each size fraction. The size-fractioned approach enabled us to highlight these differences between the size fraction biomass dynamics that would have gone unnoticed if considering the whole biomass. This revealed the strong link between the biomass size-fraction dynamic and community size structure.

The different community level indicators (i.e. size-fraction biomasses, total zooplankton abundance, size- and diversity- structure) used in our study highlighted shifts occurring in different years (Figs 4 and 6) denoting structural changes that would not have been perceived by more aggregated variables (e.g. total abundance or total biomass). In 2013 the assemblage composition from image analysis was sufficient to explain the shift. However, a finer taxonomic reanalysis would certainly be necessary to understand the 2008 shift potentially due to changes within the calanoid group, a group with high diversity in species composition, size distribution and trophic behavior [61, 62]. In addition, such a more detailed taxonomic identification, up to separation of adult and copepodite stages, could help to better interpret the fine changes in size spectrum, highly sensitive to the underlying assemblage compositions and their associated size distribution.

Overall the multiple indicator approach used in our study helped us to better characterize and understand the zooplankton community dynamic. Pitois et al. [2] also advocated a multi-indicator approach as necessary for describing zooplankton structural changes.

### 4.2-Variations of the environmental conditions and link with zooplankton

In the present study, we aimed to understand the observed zooplankton variations within an ecosystemic framework at the study site (as shown in Fig 5) including changes in the abiotic conditions and in the lower trophic levels (micro- nano- pico-plankton). We described multiple environmental trends:

- Higher values of salinity and oxygen between 2005 and 2007 (Fig 5, trend 1). This anomaly can be interpreted as the signature of stronger offshore deep-water inflows during this period. By analyzing satellite images in those years, Mayot et al. [63] highlighted particularly high phytoplankton productivity induced by strong deep water convection events occurring over a broad area in the Provencal basin with effects extending to surrounding coastal areas, including the Bay of Marseille.
- The reduction in nutrients and particulate matter inputs observed in our time series (Fig 5, trend 1) is consistent with observations already made in the Bay of Marseille, and appears to be a general trend in French Mediterranean coastal waters [64, 65]. The physical and chemical characteristics of the Bay of Marseille might have been impacted by the diminution of the Rhône nutrient inputs in the Gulf of Lion [66] and/or by the proximity to the Marseille sewage treatment plant where the treatment efficiency has improved since 2008 [32, 67]. While ammonium, nitrite and phosphate decreased, nitrate concentration was stabilized (and even presented a slight increase in the second part of the study, see S3 File). As autotrophic planktonic groups show preferences in nutrient uptake [68], the variations in the stoichiometry of nutrient sources and their concentrations may have affected their distribution.

Our observations evidenced clear changes in size and abundance of the micro-, nano-, pico-plankton in 2013 which were concomitant with changes in the mesozooplankton community structure (Figs 5B and 6B and 6C). This supports the hypothesis of a regime shift in the whole pelagic food web through bottom-up cascading effects [69, 70], similarly to those described in Rietkerk et al. [71]. The concomitant decline of calanoid copepods and increase in appendicularians and salps represented an indicator of such change in the zooplankton community. Because appendicularians and salps can feed on smaller particles, the trophic competition with calanoid copepods (feeding on larger particles) seemed to be reduced [72, 73]. When copepods are unable to feed on large microphytoplankton, they often switch to microzooplankton [74, 75] resulting in a higher trophic level of copepods [69]. The hypothesis of such a relationship between the phytoplankton size and zooplankton assemblages has been suggested in the NW Mediterranean Sea [54, 76]. These changes in the Bay of Marseille were accompanied with the appearance of predators such as chaetognaths or cnidarians. In the Catalan Sea, the predation impact of chaetognaths on the standing stock of copepod was low, but chaetognaths can exert a high level of predation on specific copepod species and stages under food-limited conditions [77]. A species level taxonomy would help to quantify the top-down pressure of predators on copepods species dynamics.

In parallel, by analyzing the winter environmental conditions, we observed a relationship between NAO, precipitation, temperature, and zooplankton phenology (Fig 9). Although at Mediterranean scale no relationship was found between NAO and zooplankton by Berline et al. [5], several studies suggested that winter conditions at NW Mediterranean Sea regional scale (inducing strong variation in the NAO index) impacted the following zooplankton spring peak [4, 27, 28], as shown by our results (and discussed in the previous section). Since 2014, winter deep convection (associated with climatic forcing NAO) declined in the NW Mediterranean Sea causing a stratification and water warming [78]. This was concomitant with the increase of winter temperature, NAO and precipitation in the Bay of Marseille, and (i) a later seasonal onset of the 200–1000 μm zooplankton biomass, copepods (calanoids and ergasilida), appendicularians, total zooplankton (ii) an earlier seasonal onset of salps. Therefore, we cannot exclude the hypothesis that winter large-scale forcing impacted the coastal zooplankton dynamics in the Bay of Marseille in the 2010s.

### 4.3-Implications of the variations in the pelagic ecosystem

**Fig 10:**
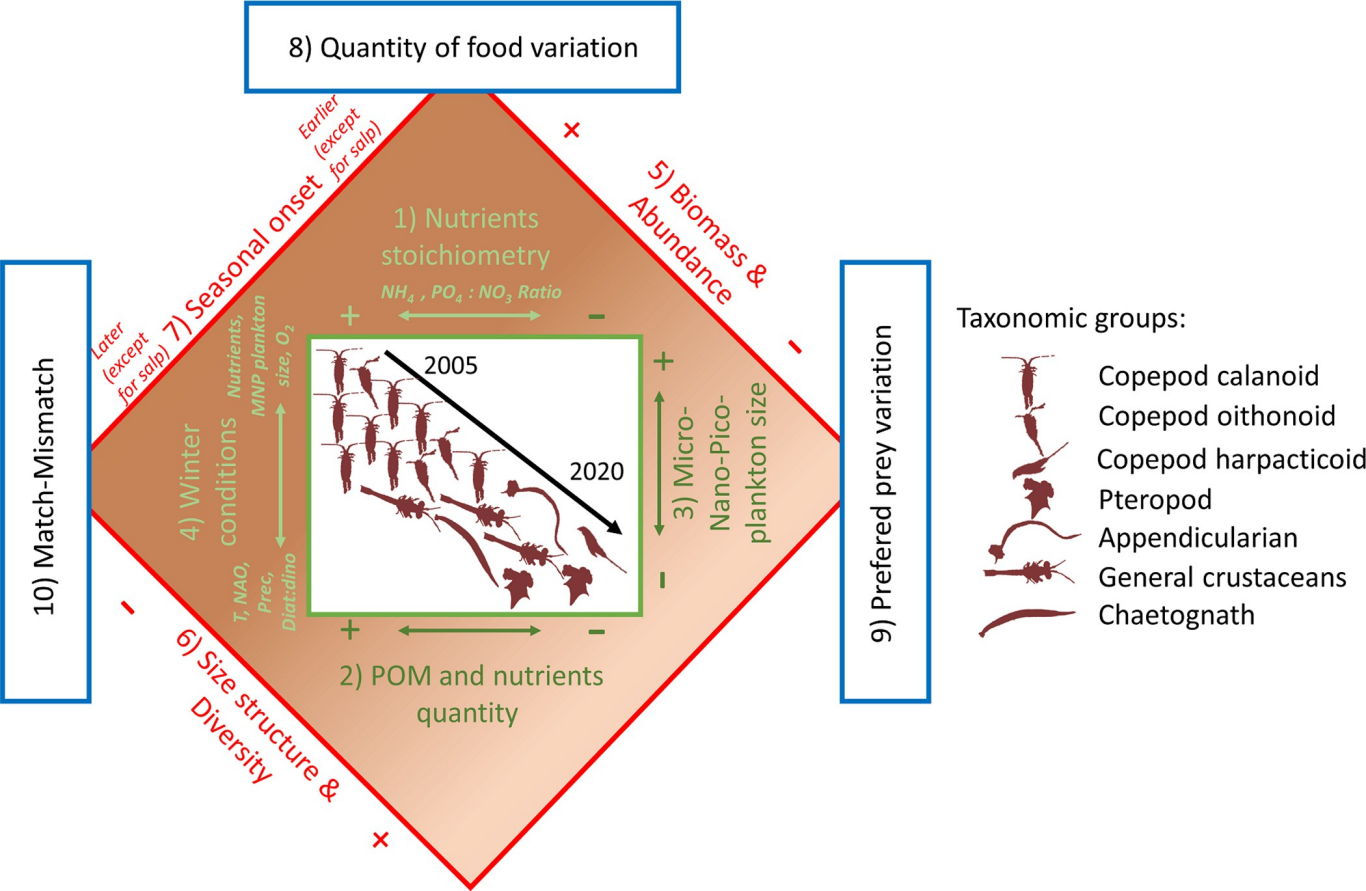
Summary of our main results by means of the revisited Ramon Margalef mandala [80]. In the schema, the significant interannual changes of the zooplankton community and environmental parameters are represented along the timeline materialized by the black arrow. The variables around the green square exerted a direct or indirect bottom-up control on the interannual zooplankton community dynamic: (1) Nutrients stoichiometry, (2) POM and nutrients quantity, (3) Micro-, Nano-, Pico- plankton size, (4) Winter conditions. The taxonomic groups within in the central square highlight the main variations of the zooplankton community composition. The variations in the zooplankton community traits are represented around the red square: (5) Biomass and abundance, (6) Size structure and diversity, (7) Seasonal onset. Finally, the implications for planktivorous fish are represented within the blue squares: (8) Quantity of food variation, (9) Preferred prey variation, (10) Match-Mismatch.

The conceptual schema, Fig 10, summarizes the interannual changes analyzed in the present paper concerning the environment of the Bay of Marseille and its mesozooplankton community. The results of the Bay of Marseille time series contribute to the documentation of the alterations in the Mediterranean pelagic ecosystem [79], particularly changes occurring in the Gulf of Lion. Espinasse et al. [17], on the basis of a large-scale survey in winter and spring, defined three zooplankton habitats in the Gulf of Lion: the continental shelf, the Rhone influence zone and the Occitan littoral zone. They found that the zooplankton habitat in the BoM presented the same environmental and biological characteristics as most of the Gulf of Lion continental shelf. Therefore, the Bay of Marseille time series can be considered as a sentinel zone for monitoring environmental effects from the open sea as well as the coastal area.

The observed sudden diminution of the larger mesozooplankton size fraction biomass (shown in Fig 4) coincided with the shift in fish body condition at Gulf of Lion scale evidenced in 2008 [20] and with observed changes in the diets of small pelagic fishes [19]. This supports the hypothesis of a bottom-up control of the pelagic food chain up to the planktivorous fishes in the Gulf of Lion [24, 66], as shown in other areas in the Mediterranean Sea [81, 82].

By taking into account indicators related to the zooplankton populations seasonal onset, we show that the process of match/mismatch with small pelagic fish [83] may be a process at work in the Gulf of Lion. An increased mismatch between the spring peak of zooplankton biomass (mainly calanoids) and the growth phase of small pelagic fish [84–86] could certainly explain their body condition. In their experimental study, Queiros et al. [87], showed that sardine can display adaptative phenotypic plasticity to food condition changes. Under lower food quantity and quality conditions, the smallest phenotypes experienced lower mortality by starvation than the larger ones during the critical post-reproductive period (i.e. at the end of winter). In this context, a delay in the availability of these preferred fish prey might favor smaller fish phenotypes. In addition, the zooplankton biochemical composition and energy content may vary seasonally [60, 88] due to the reorganization of different zooplankton assemblages. Further investigations of the interannual variations of the mesozooplankton quality, both in species composition and biochemical content, are needed to improve our understanding of changes in the trophic environment of the small pelagic fishes.

## Supporting information

S1 File Supplementary information on environmental and zooplankton monitoring. (DOCX)

S2 File Performance of ParticleTrieur software for the detection of individuals. (DOCX)

S3 File Summary of the results of the dynamic factor analysis. (DOCX)

S4 File Seasonality assessment. (DOCX)

## References

[1] Beaugrand G. Monitoring pelagic ecosystems using plankton indicators. ICES J Mar Sci. 2005;62(3):333‑8.

[2] Pitois SG, Graves CA, Close H, Lynam C, Scott J, Tilbury J, et al. A first approach to build and test the Copepod Mean Size and Total Abundance (CMSTA) ecological indicator using in-situ size measurements from the Plankton Imager (PI). Ecol Indic. 2021;123:107307.

[3] Planque B, Taylor AH. Long-term changes in zooplankton and the climate of the North Atlantic. ICES J Mar Sci. 1998;55(4):644‑54.

[4] García-Comas C, Stemmann L, Ibanez F, Berline L, Mazzocchi MG, Gasparini S, et al. Zooplankton long-term changes in the NW Mediterranean Sea: Decadal periodicity forced by winter hydrographic conditions related to large-scale atmospheric changes? J Mar Syst. 2011;87(3):216‑26.

[5] Berline L, Siokou-Frangou I, Marasović I, Vidjak O, Fernández de Puelles ML, Mazzocchi MG, et al. Intercomparison of six Mediterranean zooplankton time series. Prog Oceanogr. 2012;97‑100:76‑91.

[6] Fanjul A, Villate F, Uriarte I, Iriarte A, Atkinson A, Cook K. Zooplankton variability at four monitoring sites of the Northeast Atlantic Shelves differing in latitude and trophic status. J Plankton Res. 2017;39(6):891‑909.

[7] Hays GC, Richardson AJ, Robinson C. Climate change and marine plankton. Trends Ecol Evol. 2005;20(6):337‑44. doi: 10.1016/j.tree.2005.03.004 16701390

[8] Mackas DL, Greve W, Edwards M, Chiba S, Tadokoro K, Eloire D, et al. Changing zooplankton seasonality in a changing ocean: Comparing time series of zooplankton phenology. Prog Oceanogr. 2012;97‑100:31‑62.

[9] Pagano M, Gaudy R, Thibault D, Lochet F. Vertical Migrations and Feeding Rhythms of Mesozooplanktonic Organisms in the Rhône River Plume Area (North-west Mediterranean Sea). Estuar Coast Shelf Sci. 1993;37(3):251‑69.

[10] Razouls C, Kouwenberg J. Spatial distribution and seasonal variation of mesozooplankton biomass in the Gulf of Lions (northwestern Mediterranean). Oceanol Acta. 1993;(16):393‑401.

[11] Champalbert G. Characteristics of zooplankton standing stock and communities in the Western Mediterranean Sea: Relations to hydrology. Sci Mar. 1996;60:97‑113.

[12] Gaudy R, Champalbert G. Space and time variations in zooplankton distribution south of Marseilles. Oceanol Acta. 1998;21(6):793‑802.

[13] Plounevez S, Champalbert G. Diet, feeding behaviour and trophic activity of the anchovy (Engraulis encrasicolus L.) in the Gulf of Lions (Mediterranean Sea). Oceanol Acta. 2000;23(2):175‑92.

[14] Gaudy R, Youssara F, Diaz F, Raimbault P. Biomass, metabolism and nutrition of zooplankton in the Gulf of Lions (NW Mediterranean). Oceanol Acta. 2003;26(4):357‑72.

[15] Raimbault P, Durrieu de Madron X. Research activities in the Gulf of Lion (NW Mediterranean) within the 1997–2001 PNEC project. Oceanol Acta. 2003;26(4):291‑8.

[16] Palomera I, Olivar MP, Salat J, Sabatés A, Coll M, García A, et al. Small pelagic fish in the NW Mediterranean Sea: An ecological review. Prog Oceanogr. 2007;74(2‑3):377‑96.

[17] Espinasse B, Carlotti F, Zhou M, Devenon JL. Defining zooplankton habitats in the Gulf of Lion (NW Mediterranean Sea) using size structure and environmental conditions. Mar Ecol Prog Ser. 2014;506:31‑46.

[18] Saiz E, Sabatés A, Gili JM. The Zooplankton. In: Goffredo S, Dubinsky Z, éditeurs. The Mediterranean Sea. Dordrecht: Springer Netherlands; 2014 p. 183‑211. https://link.springer.com/doi: 10.1007/978-94-007-6704-1_11

[19] Bourg BL. Trophic niche overlap of sprat and commercial small pelagic teleosts in the Gulf of Lions (NW Mediterranean Sea). J Sea Res. 2015;9.

[20] Van Beveren E, Bonhommeau S, Fromentin JM, Bigot JL, Bourdeix JH, Brosset P, et al. Rapid changes in growth, condition, size and age of small pelagic fish in the Mediterranean. Mar Biol. 2014;161(8):1809‑22.

[21] Bănaru D, Mellon-Duval C, Roos D, Bigot JL, Souplet A, Jadaud A, et al. Trophic structure in the Gulf of Lions marine ecosystem (north-western Mediterranean Sea) and fishing impacts. J Mar Syst. 2013;111‑112:45‑68.

[22] Van Beveren E, Fromentin JM, Bonhommeau S, Nieblas AE, Metral L, Brisset B, et al. Predator–prey interactions in the face of management regulations: changes in Mediterranean small pelagic species are not due to increased tuna predation. Can J Fish Aquat Sci. 2017;74(9):1422‑30.

[23] GFCM. Scientific Advisory Committee on Fisheries (SAC) Working Group on Stock Assessment of Small Pelagic species (WGSASP). 2017.

[24] Saraux C, Van Beveren E, Brosset P, Queiros Q, Bourdeix JH, Dutto G, et al. Small pelagic fish dynamics: A review of mechanisms in the Gulf of Lions. Deep Sea Res Part II Top Stud Oceanogr. 2019;159:52‑61.

[25] Brosset P, Le Bourg B, Costalago D, Bănaru D, Van Beveren E, Bourdeix J, et al. Linking small pelagic dietary shifts with ecosystem changes in the Gulf of Lions. Mar Ecol Prog Ser. 2016;554:157‑71.

[26] Serranito B, Jamet JL, Rossi N, Jamet D. Decadal shifts of coastal microphytoplankton communities in a semi-enclosed bay of NW Mediterranean Sea subjected to multiple stresses. Estuar Coast Shelf Sci. 2019;224:171‑86.

[27] Fullgrabe L, Grosjean P, Gobert S, Lejeune P, Leduc M, Engels G, et al. Zooplankton dynamics in a changing environment: A 13-year survey in the northwestern Mediterranean Sea. Mar Environ Res. 2020;159:104962. doi: 10.1016/j.marenvres.2020.104962 32662424

[28] Fernández de Puelles ML, Alemany F, Jansá J. Zooplankton time-series in the Balearic Sea (Western Mediterranean): Variability during the decade 1994–2003. Prog Oceanogr. 2007;74(2):329‑54.

[29] Millot C. Wind induced upwellings in the gulf of lions. Oceanol Acta. 1979;2(3):261‑74.

[30] Pinazo C, Fraysse M, Doglioli A, Faure VM, Pairaud I, Petrenko A, et al. MASSILIA: Modélisation de la baie de MArSeILLe: Influence des apports Anthropiques de la métropole sur l’écosystème marin. 2013; https://archimer.ifremer.fr/doc/00145/25592/

[31] Millot C. Circulation in the Western Mediterranean Sea. J Mar Syst. 1999;20(1):423‑42.

[32] Millet B, Pinazo C, Banaru D, Pagès R, Guiart P, Pairaud I. Unexpected spatial impact of treatment plant discharges induced by episodic hydrodynamic events: Modelling Lagrangian transport of fine particles by Northern Current intrusions in the bays of Marseille (France). PLOS ONE. 2018;13(4):e0195257. doi: 10.1371/journal.pone.0195257 29694362 PMC5918620

[33] Gregoire D. France Geojson. 2018. https://github.com/gregoiredavid/france-geojson

[34] Martin-Vide J, Lopez-Bustins JA. The Western Mediterranean Oscillation and rainfall in the Iberian Peninsula. Int J Climatol. 2006;26(11):1455‑75.

[35] Quéguiner B, Carlotti F, Leblanc K, Salter I, Golbol M, Guilloux L, et al. Mistrals/Specimed Project: Seasonal And Interannual Variability Of Plankton Communities Structure And Biogeochemical Cycles In North-Western Mediterranean. In 2013.

[36] Phytobs. PHYTOBS dataset ‐ French National Service of Observation for Phytoplankton in coastal waters SEANOE; 2021. https://www.seanoe.org/data/00740/85178/

[37] Gorsky G, Ohman MD, Picheral M, Gasparini S, Stemmann L, Romagnan JB, et al. Digital zooplankton image analysis using the ZooScan integrated system. J Plankton Res. 2010;32(3):285‑303.

[38] Jennings BR, Parslow K, Ottewill RH. Particle size measurement: the equivalent spherical diameter. Proc R Soc Lond Math Phys Sci. 1988;419(1856):137‑49.

[39] Marchant R, Tetard M, Pratiwi A, Adebayo M, de Garidel-Thoron T. Automated analysis of foraminifera fossil records by image classification using a convolutional neural network. J Micropalaeontology. 2020;39(2):183‑202.

[40] R Core Team. R: A Language and Environment for Statistical Computing. Vienna, Austria: R Foundation for Statistical Computing; 2022. https://www.R-project.org/

[41] Tukey JW (John W. Exploratory data analysis [Internet]. Reading, Mass.: Addison-Wesley Pub. Co.; 1977. 714 p. http://archive.org/details/exploratorydataa0000tuke_7616

[42] Nerini D, Ghattas B. Classifying densities using functional regression trees: Applications in oceanology. Comput Stat Data Anal. 2007;51(10):4984‑93.

[43] Ramsay JO, Hooker G, Graves S. Introduction to Functional Data Analysis. In: Ramsay J, Hooker G, Graves S, éditeurs. Functional Data Analysis with R and MATLAB [Internet]. New York, NY: Springer; 2009 p. 1‑19. (Use R). 10.1007/978-0-387-98185-7_1

[44] Pauthenet E, Roquet F, Madec G, Nerini D. A Linear Decomposition of the Southern Ocean Thermohaline Structure. J Phys Oceanogr. 2017;47(1):29‑47.

[45] Dixon P. VEGAN, a package of R functions for community ecology. J Veg Sci. 2003;14(6):927‑30.

[46] Audigier V, Husson F, Josse J. Multiple imputation for continuous variables using a Bayesian principal component analysis. arXiv; 2015. http://arxiv.org/abs/1401.5747

[47] Zeileis A, Leisch F, Hornik K, Kleiber C. strucchange: An R Package for Testing for Structural Change in Linear Regression Models. J Stat Softw. 2002;7:1‑38.

[48] Greve W, Lange U, Reiners F, Nast J. Predicting the seasonality of North Sea zooplankton. Senckenberg Maritima. 2001;31(2):263‑8.

[49] Zuur AF, Fryer RJ, Jolliffe IT, Dekker R, Beukema JJ. Estimating common trends in multivariate time series using dynamic factor analysis. Environmetrics. 2003;14(7):665‑85.

[50] Holmes E E, Ward E J, Wills K. MARSS: Multivariate Autoregressive State-space Models for Analyzing Time-series Data. R J. 2012;4(1):11.

[51] Fernández de Puelles ML, Macias V, Vicente L, Molinero JC. Seasonal spatial pattern and community structure of zooplankton in waters off the Baleares archipelago (Central Western Mediterranean). J Mar Syst. 2014;138:82‑94.

[52] Romagnan JB, Legendre L, Guidi L, Jamet JL, Jamet D, Mousseau L, et al. Comprehensive Model of Annual Plankton Succession Based on the Whole-Plankton Time Series Approach. PLOS ONE. 2015;10(3):e0119219. doi: 10.1371/journal.pone.0119219 25780912 PMC4363592

[53] García-Martínez M del C, Vargas-Yáñez M, Moya F, Santiago R, Reul A, Muñoz M, et al. Spatial and Temporal Long-Term Patterns of Phyto and Zooplankton in the W-Mediterranean: RADMED Project. Water. 2019;11(3):534.

[54] Feuilloley G, Fromentin J marc, Saraux C, Irisson JO, Jalabert L, Stemmann L. Temporal fluctuations in zooplankton size, abundance, and taxonomic composition since 1995 in the North Western Mediterranean Sea. ICES J Mar Sci 2021; https://hal.archives-ouvertes.fr/hal-03374991

[55] Irigoien X, Huisman J, Harris RP. Global biodiversity patterns of marine phytoplankton and zooplankton. Nature. juin 2004;429(6994):863‑7. doi: 10.1038/nature02593 15215862

[56] Donoso K, Carlotti F, Pagano M, Hunt BPV, Escribano R, Berline L. Zooplankton community response to the winter 2013 deep convection process in the NW Mediterranean Sea. J Geophys Res Oceans. 2017;122(3):2319‑38.

[57] Berline L, Rammou AM, Doglioli A, Molcard A, Petrenko A. A Connectivity-Based Eco-Regionalization Method of the Mediterranean Sea. PLOS ONE. 2014;9(11):e111978. doi: 10.1371/journal.pone.0111978 25375212 PMC4222956

[58] Myers RA. When Do Environment–recruitment Correlations Work? Rev Fish Biol Fish. 1998;8(3):285‑305.

[59] Bănaru D, Carlotti F, Barani A, Grégori G, Neffati N, Harmelin-Vivien M. Seasonal variation of stable isotope ratios of size-fractionated zooplankton in the Bay of Marseille (NW Mediterranean Sea). J Plankton Res. 2014;36(1):145‑56.

[60] Chen CT, Bănaru D, Carlotti F, Faucheux M, Harmelin-Vivien M. Seasonal variation in biochemical and energy content of size-fractionated zooplankton in the Bay of Marseille (North-Western Mediterranean Sea). J Mar Syst. 2019;199:103223.

[61] Kleppel G. On the diets of calanoid copepods. Mar Ecol Prog Ser. 1993;99:183‑95.

[62] Beaugrand G, Reid P, Ibañez F, Planque B. Biodiversity of North Atlantic and North Sea calanoid copepods. Mar Ecol Prog Ser. 2000;204:299‑303.

[63] Mayot N, D’Ortenzio F, Uitz J, Gentili B, Ras J, Vellucci V, et al. Influence of the Phytoplankton Community Structure on the Spring and Annual Primary Production in the Northwestern Mediterranean Sea. J Geophys Res Oceans. 2017;122(12):9918‑36.

[64] Lheureux A, Savoye N, Amo YD, Goberville E, Bozec Y, Breton E, et al. Bi-decadal variability in physico-biogeochemical characteristics of temperate coastal ecosystems: from large-scale to local drivers. Mar Ecol Prog Ser. 2021;660:19‑35.

[65] Goberville E, Beaugrand G, Sautour B, Tréguer P, Team S. Climate-driven changes in coastal marine systems of western Europe. Mar Ecol Prog Ser. 2010;408:129‑48.

[66] Feuilloley G, Fromentin JM, Stemmann L, Demarcq H, Estournel C, Saraux C. Concomitant changes in the environment and small pelagic fish community of the Gulf of Lions. Prog Oceanogr. 2020;186:102375.

[67] Raimbault P, Boudouresque CF, Bănaru D, Jacquet S, Thibault D, Vincente N, et al. Chapitre 7: Le milieu marin autour de Marseille. In: Curt T, Guiot J, Mazurek H, éditeurs. Marseille et l’environnement Bilan, qualité et enjeux: Le développemennt durable d’une grande ville littorale face au changement climatique. Aix-en-Provence: Presses universitaires de Provence; 2021. p. 171‑219. (Hors collection). http://books.openedition.org/pup/41463

[68] Probyn TA, Painting SJ. Nitrogen uptake by size-fractionated phytoplankton populations in Antarctic surface waters1. Limnol Oceanogr. 1985;30(6):1327‑32.

[69] Sommer U, Stibor H, Katechakis A, Sommer F, Hansen T. Pelagic food web configurations at different levels of nutrient richness and their implications for the ratio fish production:primary production. In: Vadstein O, Olsen Y, éditeurs. Sustainable Increase of Marine Harvesting: Fundamental Mechanisms and New Concepts: Proceedings of the 1st Maricult Conference held in Trondheim, Norway, 25–28 June 2000. Dordrecht: Springer Netherlands; 2002. p. 11‑20. 10.1007/978-94-017-3190-4_2

[70] Stibor H, Vadstein O, Diehl S, Gelzleichter A, Hansen T, Hantzsche F, et al. Copepods act as a switch between alternative trophic cascades in marine pelagic food webs. Ecol Lett. 2004;7(4):321‑8.

[71] Rietkerk M, Dekker SC, de Ruiter PC, van de Koppel J. Self-Organized Patchiness and Catastrophic Shifts in Ecosystems. Science. 2004;305(5692):1926‑9. doi: 10.1126/science.1101867 15448261

[72] Flood PR, Deibel D, Morris CC. Filtration of colloidal melanin from sea water by planktonic tunicates. Nature. 1992;355(6361):630‑2.

[73] Katechakis A, Stibor H, Sommer U, Hansen T. Feeding selectivities and food niche separation of Acartia clausi, Penilia avirostris (Crustacea) and Doliolum denticulatum (Thaliacea) in Blanes Bay (Catalan Sea, NW Mediterranean). J Plankton Res. 2004;26(6):589‑603.

[74] Stoecker DK, Capuzzo JM. Predation on Protozoa: its importance to zooplankton. J Plankton Res. 1990;12(5):891‑908.

[75] Irigoien X, Head RN, Harris RP, Cummings D, Harbour D, Meyer-Harms B. Feeding selectivity and egg production of Calanus helgolandicus in the English Channel. Limnol Oceanogr. 2000;45(1):44‑54.

[76] Calbet A, Garrido S, Saiz E, Alcaraz M, Duarte CM. Annual Zooplankton Succession in Coastal NW Mediterranean Waters: The Importance of the Smaller Size Fractions. J Plankton Res. 2001;23(3):319‑31.

[77] Duró A, Saiz E. Distribution and trophic ecology of chaetognaths in the western Mediterranean in relation to an inshore–offshore gradient. J Plankton Res. 2000;22(2):339‑61.

[78] Margirier F, Testor P, Heslop E, Mallil K, Bosse A, Houpert L, et al. Abrupt warming and salinification of intermediate waters interplays with decline of deep convection in the Northwestern Mediterranean Sea. Sci Rep. 2020;10(1):20923. doi: 10.1038/s41598-020-77859-5 33262416 PMC7708500

[79] Durrieu de Madron X, Guieu C, Sempéré R, Conan P, Cossa D, D’Ortenzio F, et al. Marine ecosystems’ responses to climatic and anthropogenic forcings in the Mediterranean. Prog Oceanogr. 2011;91(2):97‑166.

[80] Glibert PM. Margalef revisited: A new phytoplankton mandala incorporating twelve dimensions, including nutritional physiology. Harmful Algae. 2016;55:25‑30. doi: 10.1016/j.hal.2016.01.008 28073538

[81] Macias D, Garcia-Gorriz E, Piroddi C, Stips A. Biogeochemical control of marine productivity in the Mediterranean Sea during the last 50 years. Glob Biogeochem Cycles. 2014;28(8):897‑907. doi: 10.1002/2014GB004846 26180286 PMC4493898

[82] Piroddi C, Coll M, Liquete C, Macias D, Greer K, Buszowski J, et al. Historical changes of the Mediterranean Sea ecosystem: modelling the role and impact of primary productivity and fisheries changes over time. Sci Rep. 2017;7(1):44491. doi: 10.1038/srep44491 28290518 PMC5349533

[83] Cushing DH. Plankton Production and Year-class Strength in Fish Populations: an Update of the Match/Mismatch Hypothesis. In: Blaxter JHS, Southward AJ, éditeurs. Advances in Marine Biology. Academic Press; 1990. p. 249‑93. https://www.sciencedirect.com/science/article/pii/S0065288108602023

[84] Costalago D, Palomera I. Feeding of European pilchard (Sardina pilchardus) in the northwestern Mediterranean: from late larvae to adults. Sci Mar. 2014;78(1):41‑54.

[85] Nikolioudakis N, Isari S, Somarakis S. Trophodynamics of anchovy in a non-upwelling system: direct comparison with sardine. Mar Ecol Prog Ser. 2014;500:215‑29.

[86] Chen CT, Carlotti F, Harmelin-Vivien M, Guilloux L, Bănaru D. Temporal variation in prey selection by adult European sardine (Sardina pilchardus) in the NW Mediterranean Sea. Prog Oceanogr. 2021;196:102617.

[87] Queiros Q, Saraux C, Dutto G, Gasset E, Marguerite A, Brosset P, et al. Is starvation a cause of overmortality of the Mediterranean sardine? Mar Environ Res. 2021;170:105441. doi: 10.1016/j.marenvres.2021.105441 34411887

[88] Barroeta Z, Olivar MP, Palomera I. Energy density of zooplankton and fish larvae in the southern Catalan Sea (NW Mediterranean). J Sea Res. 2017;124:1‑9.

