# Supplementary material for: Temporal changes in zooplankton indicators highlight a bottom-up process in the Bay of Marseille (NW Mediterranean Sea)
Source: PLoS One. 2023 Oct 23;18(10):e0292536. doi: 10.1371/journal.pone.0292536 (PMC10593231; doi:10.1371/journal.pone.0292536)
Supplement: S1 File — (DOCX) [file pone.0292536.s001.docx]

# **Supplementary information on environmental and zooplankton monitoring.**

1. **Environmental monitoring**

Twice a month since 1994, in addition to a CTD-oxygen-fluorometer cast delivering temperature (T, in °C), salinity (S, measured in practical salinity unit), oxygen concentration (O_2_, in mL.L^-1^) throughout the water column (0-55m depth), the following environmental variables were measured at three depths (surface, bottom and fluorescence maximum) by means of Niskin bottles: nutrient concentrations (in µmol.L^-1^) such as ammonium (NH_4_), nitrate (NO_3_), nitrite (NO_2_), phosphate (PO_4_); concentrations (in µg.L^-1^) of particulate organic carbon (POC), particulate organic nitrogen (PON), suspended particulate matter (SPM) and chlorophyll *a* (CHLA). In the present study, the measurement values for each environmental variable obtained at the three depths were averaged.

Microphytoplankton was also collected at the surface with a dedicated Niskin bottle. Microphytoplankton taxa were counted by a taxonomist by optical microscopy, in the context of the SPECIMED [1] project and PHYTOBS program [2] respectively during 2010-2013 and since 2016, and were aggregated in two functional groups: diatoms and dinoflagellates. The ratio of log abundances of diatoms and dinoflagellates was calculated to picture the community structure of microphytoplankton. Additionally, samples from the surface were also collected from October 2009 for cytometry analysis delivering counts (cells.mL^-1^) and size index for three heterotrophic prokaryotic groups (high nucleic acid, low nucleic acid, and total bacteria cells), and 5 groups of pico- and nano-phytoplankton : *Cryptophycea*, *Prochlorococcus*, *Synechococcus*, pico- and nano- eucaryotes.

From temperature and salinity profiles and depth, water density was calculated [3] . The Mixed Layer Depth (MLD) was calculated as the depth that presents a difference of 0.03 Kg.m^-3^ from the 10 m depth density, according to the method described in [4]. Precipitations and wind stress time series were obtained from Météo France (<https://donneespubliques.meteofrance.fr/>). Positive and negative wind stress values illustrate respectively north-westerly (Mistral) and south-easterly winds. Both Northern Atlantic Oscillations (NAO, <https://www.ncdc.noaa.gov/teleconnections/nao/>) and Western Mediterranean Oscillations (WeMO, <https://crudata.uea.ac.uk/cru/data/moi/>) data series were compiled to study large-scale variations of NW Mediterranean atmospheric conditions.

**2) Zooplankton monitoring**

In parallel with the SOMLIT monitoring, mesozooplankton samples were collected since 2005 through vertical haul using a WP2 200 µm mesh size plankton net. The samples were fractioned in two. The first half of the cod-end content was preserved in a 4% buffered formaldehyde solution, the other half was maintained in cold condition for further laboratory treatment. We are aware that smaller mesozooplankton taxa might not be perfectly sampled by the WP2 200 µm mesh size net [5]. Notwithstanding, this net captures the main zooplankton prey size classes consumed by planktivorous teleosts in this area [6,7] and therefore enables us to picture the main mode of changes of the mesozooplankton community structure [8] .

# **References of supplementary material.**

1. Quéguiner B, Carlotti F, Leblanc K, Salter I, Golbol M, Guilloux L, et al. MISTRALS/SPECIMED Project: Seasonal and Interannual Variability of Plankton Communities structure and biogeochemical cycles in North-Western Mediterranean. In 2013.

2. Phytobs. PHYTOBS dataset - French National Service of Observation for Phytoplankton in coastal waters. SEANOE; 2021. https://www.seanoe.org/data/00740/85178/

3. Fofonoff NP. Algorithms for computation of fundamental properties of seawater. Paris, France; 1983 p. 53. (UNESCO technical papers in marine science). Report No.: 44. https://doi.org/10.25607/OBP-1450

4. de Boyer Montégut C, Madec G, Fischer AS, Lazar A, Iudicone D. Mixed layer depth over the global ocean: An examination of profile data and a profile-based climatology. J Geophys Res Oceans. 2004;109(C12). https://onlinelibrary.wiley.com/doi/abs/10.1029/2004JC002378

5. Batten SD, Clark R, Flinkman J, Hays G, John E, John AWG, et al. CPR sampling: the technical background, materials and methods, consistency and comparability. Prog Oceanogr. 2003;58(2):193‑215.

6. Le Bourg B, Bănaru D, Saraux C, Nowaczyk A, Le Luherne E, Jadaud A, et al. Trophic niche overlap of sprat and commercial small pelagic teleosts in the Gulf of Lions (NW Mediterranean Sea). J Sea Res. 2015;103:138‑46.

7. Chen CT, Carlotti F, Harmelin-Vivien M, Guilloux L, Bănaru D. Temporal variation in prey selection by adult European sardine (*Sardina pilchardus*) in the NW Mediterranean Sea. Prog Oceanogr. 2021;196:102617.

8. Peck LS. Ecophysiology of Antarctic marine ectotherms: limits to life. In: Arntz WE, Clarke A, éditeurs. Ecological Studies in the Antarctic Sea Ice Zone: Results of EASIZ Midterm Symposium. Berlin, Heidelberg: Springer; 2002. p. 221‑30. https://doi.org/10.1007/978-3-642-59419-9_29
