# Supplementary material for: Temporal changes in zooplankton indicators highlight a bottom-up process in the Bay of Marseille (NW Mediterranean Sea)
Source: PLoS One. 2023 Oct 23;18(10):e0292536. doi: 10.1371/journal.pone.0292536 (PMC10593231; doi:10.1371/journal.pone.0292536)
Supplement: S2 File — (DOCX) [file pone.0292536.s002.docx]

# **Performance of ParticleTrieur software for the detection of individuals.**

**Zooplankton digitalization procedure**

The aliquot conserved in the formaldehyde solution was used for plankton digitalization. It was firstly fractionated in two size-fractions with sieves of 1000 µm (referred to below as fraction >1000 µm or fraction <1000 µm). Then, each fraction was split into two equal parts with a Motoda box, and this procedure was repeated until an adequate quantity of organisms was obtained to be processed with the Zooscan in order to scan about 1,500 individuals correctly separated on the scan window, and then digitalized with one image (vignette) for every individual. Because the number of repeated splits with the Motoda box for the fraction > 1000 µm is much less for the fraction <1000 µm, this procedure offers the means to avoid an under-estimation of larger organisms which would occur if the sample were treated as a whole. For the zooplankton time series, a total of 776,446 vignettes were made from 320 samples.

**Zooplankton classification with Particle Trieur**

ParticleTrieur is a software based on a convolutional neural network (CNN), trained with classified images (also called vignettes identified by an expert in taxonomy), developed to predict unclassified images. The model trained and used in this study is a neural network with 16 filters and cyclic layers. The CNN was trained and tested with 14,603 labeled images (80% for training and 20% for testing) to class images in 32 classes (3 detritus and 29 zooplankton *taxa*). Up to 14,603 images were used to train the CNN in order to identify 32 classes (see following Table 1). For every class 80% of the images were used as the training set and the remaining 20% for the classification test. The confusion matrix showed that, except for some taxa, the precision (false positive rate) and recall (false negative rate) exceed 80%, and the total accuracy of the model is 83.5% (Fig 1). This model was used for the prediction of 776,446 vignettes. Based on the performance of this model and on the (ecological/functional) similarity of some classes, we have merged some classes into categories for data analyses (third column of the table). We excluded classes that were not of interest in this work (e.g.: images of non-living objects) or with a poor rate of accurate classification (e.g. fish larvae, echinodermata larvae). From the 29 zooplankton this procedure finally led into a classification in 13 taxonomic groups.

Table 1: Summary of the class made for image classification, the number of images used for training and the group into which every class were merged to after prediction and used for data analysis. Note that classes which do not belong to a 'merged group' were not used for data analysis.

| Class | Number of images for training the CNN | Merged categories (after prediction on unknown vignettes) |
| --- | --- | --- |
| aggregats_debris | 496 | - |
| bivalvia | 254 | bivalves |
| chaetognatha | 602 | chaetognaths |
| cnidaria_and_fragments | 402 | cnidarians |
| cnidaria_siphonophorae | 305 | cnidarians |
| cop_calanoida | 840 | calanoids |
| cop_oithonoida | 776 | oithonoids |
| cop_ergasilida | 776 | ergasilida |
| cop_double | 804 | - |
| cop_fuzzy | 361 | - |
| cop_harpacticoida | 782 | harpacticoids |
| crust_amphipoda | 116 | crustaceans |
| crust_calyptopis | 112 | crustaceans |
| crust_cladocera | 28 | crustaceans |
| crust_crustacea | 500 | crustaceans |
| crust_evadne_pseudevadne | 792 | crustaceans |
| crust_ostracoda | 348 | crustaceans |
| crust_penilia | 401 | crustaceans |
| crust_podon | 183 | crustaceans |
| echinodermata_larvae | 66 | - |
| fiber | 836 | - |
| fish_eggs | 322 | fish eggs |
| fish_larvae | 19 | - |
| fuzzy_objects | 955 | - |
| nauplii | 538 | nauplii |
| polychaeta | 266 | - |
| pter_cavolinidae_cliidae | 97 | pteropods |
| pter_cavolinidae_creseidae | 262 | pteropods |
| pter_limacinidae | 243 | pteropods |
| tuni_appendicularia | 795 | appendicularians |
| tuni_appendicularia_tail | 799 | appendicularians |
| tuni_doliolida_salpida | 527 | salps |


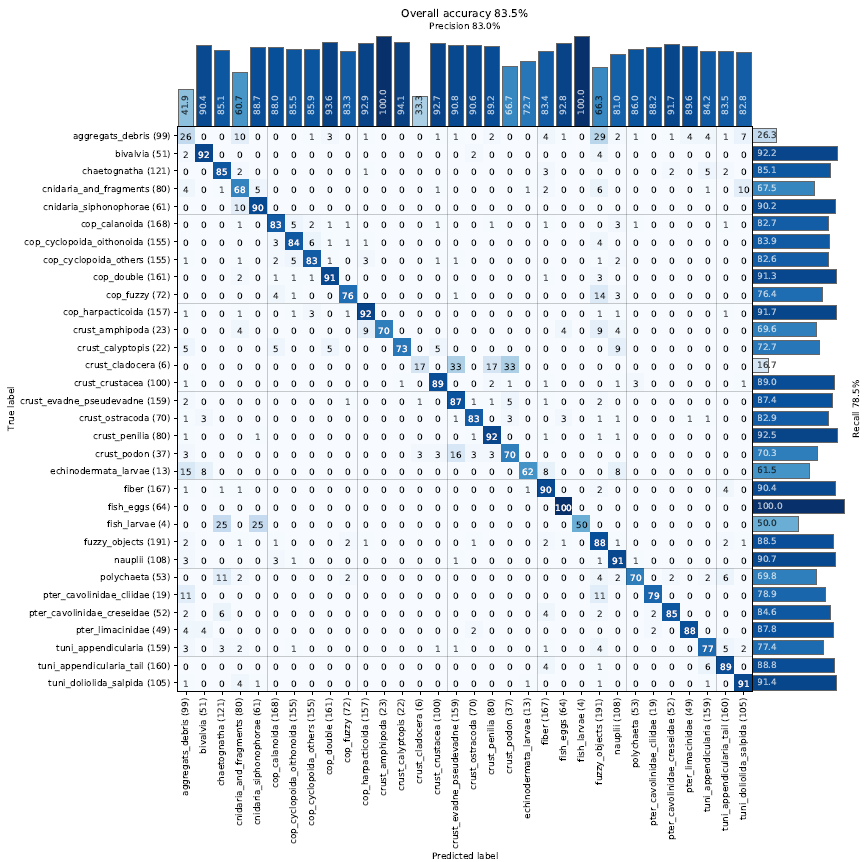


cop_oithonoida

cop_ergasilida

cop_oithonoida

cop_ergasilida

Fig 1. Confusion matrix of the test performed on the 20% remaining images of the training dataset.
