# Supplementary material for: Temporal changes in zooplankton indicators highlight a bottom-up process in the Bay of Marseille (NW Mediterranean Sea)
Source: PLoS One. 2023 Oct 23;18(10):e0292536. doi: 10.1371/journal.pone.0292536 (PMC10593231; doi:10.1371/journal.pone.0292536)
Supplement: S3 File — (DOCX) [file pone.0292536.s003.docx]

# **Summary of the results of the Dynamic Factor analysis.**

Table 1: Summary of the 12 DFA models performed on environmental data. The three first DFA model have a delta AICc within a range of 10 units, the model with the lower number of parameters was considered as the best data model (in bold).

| R matrix | Number of hidden common trends | Aikaike information Criterion | Model convergence | Number of parameters estimated |
| --- | --- | --- | --- | --- |
| diagonal and equal | 3 | 2294 | Yes | 100 |
| equalvarcov | 3 | 2296 | Yes | 101 |
| **diagonal and equal** | **2** | **2299** | **Yes** | **68** |
| equalvarcov | 2 | 2300 | Yes | 69 |
| equalvarcov | 1 | 2318 | Yes | 36 |
| diagonal and equal | 1 | 2332 | Yes | 35 |
| unconstrained | 1 | - | No | - |
| unconstrained | 2 | - | No | - |
| unconstrained | 3 | - | No | - |
| diagonal and unequal | 3 | - | No | - |
| diagonal and unequal | 2 | - | No | - |
| diagonal and unequal | 1 | - | No | - |


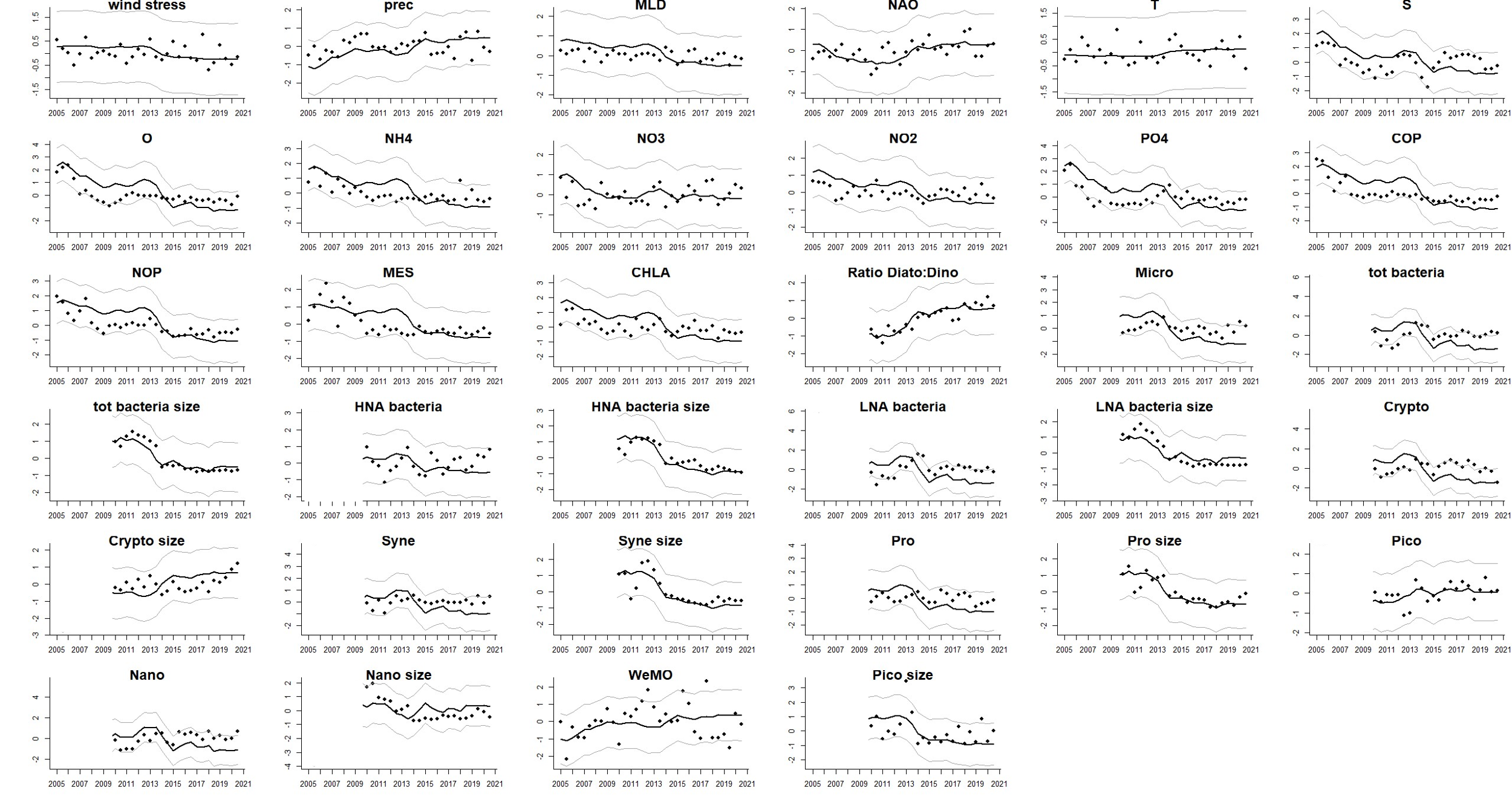


Figure 1 : Adjustment of the best DFA model (diagonal and equal R matrix and two trend) over the 2005-2020 period to the environment observational data (dots, zscore). Thick line corresponds to the mean trend and thin line correspond the 95% confidence interval

Table 2: Summary of the 12 DFA models performed on zooplankton data. The three first DFA model have a delta AICc within a range of 10 units, therefore the model with the lower number of parameters was considered as the best data model (in bold).

| R matrix | Number of hidden common trends | Aikaike information Criterion | Model convergence | Number of parameters estimated |
| --- | --- | --- | --- | --- |
| diagonal and unequal | 3 | 872.6 | Yes | 41 |
| unconstrained | 1 | 876.97 | Yes | 77 |
| **diagonal and unequal** | **2** | **877.58** | **Yes** | **32** |
| diagonal and unequal | 1 | 897.49 | Yes | 22 |
| diagonal and equal | 3 | 898.21 | Yes | 31 |
| equalvarcov | 3 | 900.4 | Yes | 32 |
| unconstrained | 2 | 904.03 | Yes | 87 |
| diagonal and equal | 2 | 905.11 | Yes | 22 |
| equalvarcov | 2 | 907.32 | Yes | 23 |
| unconstrained | 3 | 932.5 | Yes | 96 |
| diagonal and equal | 1 | 933.11 | Yes | 12 |
| equalvarcov | 1 | 934.6 | Yes | 13 |


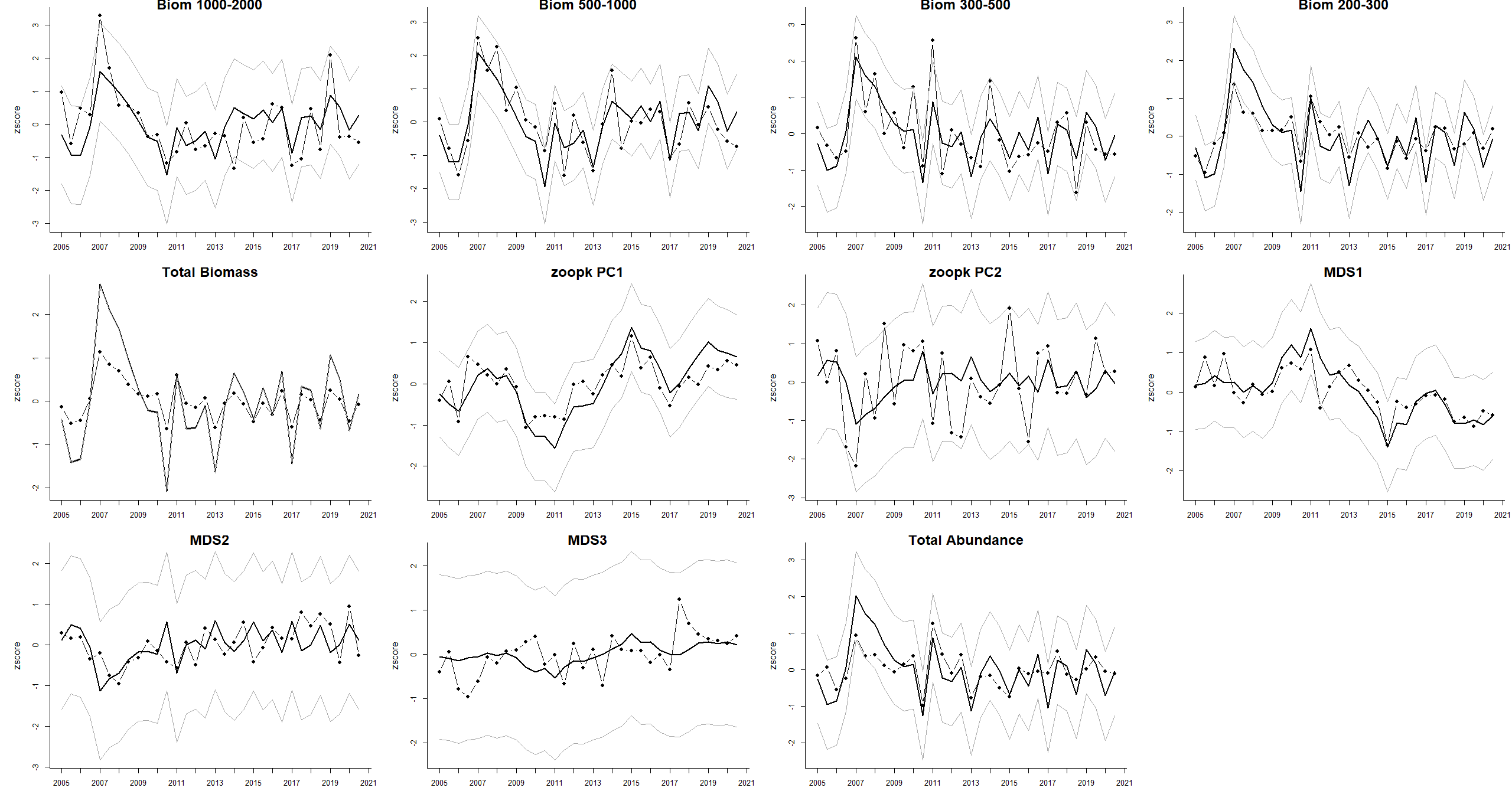


Figure 2 : Adjustment of the best DFA model (diagonal and equal R matrix and one trend and one covariate) to zooplankton the observational data (2005-2020). Thick line corresponds to the mean trend and thin line correspond the 95% confidence intervals.

Table 3: Summary of the 12 DFA models performed on winter environmental data and zooplankton phenology data. The two first DFA model have a delta AICc within a range of 10 units, therefore the model with the lower number of parameters was considered as the best data model (in bold).

| R matrix | Number of hidden common trends | Aikaike information Criterion | Model convergence | Number of parameters estimated |
| --- | --- | --- | --- | --- |
| equalvarcov | 1 | 2000 | Yes | 55 |
| **diagonal and equal** | **1** | **2003** | **Yes** | **54** |
| diagonal and unequal | 1 | 2037 | Yes | 106 |
| equalvarcov | 2 | 2043 | Yes | 107 |
| diagonal and equal | 2 | 2045 | Yes | 106 |
| diagonal and unequal | 3 | - | No | - |
| diagonal and equal | 3 | - | No | - |
| equalvarcov | 3 | - | No | - |
| unconstrained | 1 | - | No | - |
| unconstrained | 2 | - | No | - |
| unconstrained | 3 | - | No | - |
| diagonal and unequal | 2 | - | No | - |


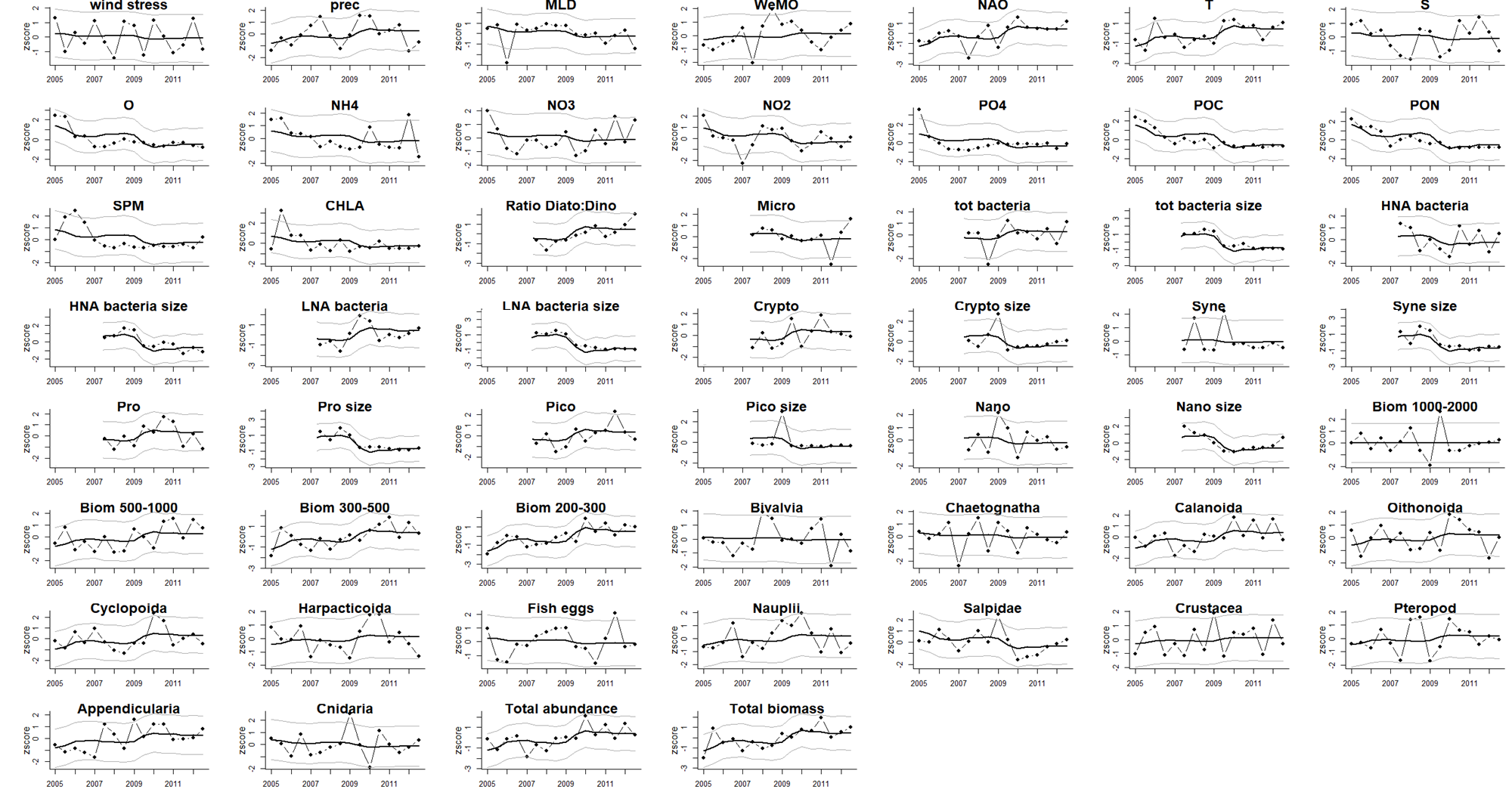


**Ergasilida**

Figure 3: Adjustment of the best DFA model (diagonal and equal with one trend) to environmental winter conditions and zooplankton seasonal onset (dots-lines, zscore). Thick line corresponds to the mean trend and thin line correspond the 95% confidence interval

| Variable  Table 4: Summary of the variable weight in the different trends and models. | Environmental DFA | | Zooplankton DFA | | Winter environment – zooplankton seasonal onset DFA |
| --- | --- | --- | --- | --- | --- |
|  | Trend 1 | Trend 2 | Trend 1 | Trend 2 | Trend 1 |
| CHLA | 0.32 | -0.07 | - | - | 0.2 |
| Cryptophycea | 0.63 | 0.06 | - | - | -0.27 |
| Cryptophycea size | -0.22 | 0.05 | - | - | 0.36 |
| HNA bacteria | 0.24 | 0.02 | - | - | 0.22 |
| HNA bacteria size | 0.12 | -0.28 | - | - | 0.55 |
| LNA bacteria | 0.65 | 0.1 | - | - | -0.35 |
| LNA bacteria size | -0.12 | -0.29 | - | - | 0.63 |
| Micro | 0.4 | -0.1 | - | - | 0.19 |
| MLD | 0.15 | -0.07 | - | - | 0.19 |
| Nano eucaryotes | 0.58 | 0.16 | - | - | 0.15 |
| Nano eucaryotes size | -0.33 | -0.23 | - | - | 0.54 |
| NAO | 0.02 | 0.15 | - | - | -0.35 |
| NH4 | 0.31 | -0.06 | - | - | 0.17 |
| NO2 | 0.22 | -0.03 | - | - | 0.26 |
| NO3 | 0.15 | 0.09 | - | - | 0.12 |
| O | 0.43 | -0.05 | - | - | 0.39 |
| Picoeukaryotes | 0.1 | 0.15 | - | - | -0.3 |
| Picoeukaryotes size | 0.25 | -0.14 | - | - | 0.3 |
| PO4 | 0.43 | 0.03 | - | - | 0.27 |
| POC | 0.38 | -0.09 | - | - | 0.43 |
| PON | 0.31 | -0.13 | - | - | 0.46 |
| prec | -0.19 | -0.01 | - | - | -0.22 |
| Prochloroccochus | 0.38 | -0.02 | - | - | -0.27 |
| Prochloroccochus size | 0.06 | -0.27 | - | - | 0.6 |
| Ratio Diato : Dino | 0.02 | 0.27 | - | - | -0.39 |
| S | 0.35 | 0.03 | - | - | 0.09 |
| SPM | 0.22 | -0.11 | - | - | 0.23 |
| Synecochoccus | 0.47 | 0.08 | - | - | 0.05 |
| Synecochoccus size | 0.13 | -0.26 | - | - | 0.56 |
| T | -0.02 | 0.03 | - | - | -0.36 |
| Total Bacteria | 0.65 | 0.1 | - | - | -0.22 |
| Total Bacteria size | -0.04 | -0.29 | - | - | 0.62 |
| WeMO | -0.17 | -0.04 | - | - | -0.09 |
| Wind stress | 0.06 | -0.05 | - | - | 0.07 |
| Appendicularia | - | - | - | - | -0.23 |
| Biom 1000-2000 | - | - | 0.71 | -0.15 | 0 |
| Biom 200-300 | - | - | 1.03 | 0.13 | -0.44 |
| Biom 300-500 | - | - | 0.93 | 0.11 | -0.33 |
| Biom 500-1000 | - | - | 0.93 | -0.16 | -0.22 |
| Bivalvia | - | - | - | - | 0.04 |
| Calanoida | - | - | - | - | -0.3 |
| Chaetognatha | - | - | - | - | 0.08 |
| Cnidaria | - | - | - | - | 0.11 |
| Crustacea | - | - | - | - | -0.08 |
| Ergasilida | - | - | - | - | -0.27 |
| Fish eggs | - | - | - | - | 0.08 |
| Harpacticoida | - | - | - | - | -0.12 |
| MDS1 | - | - | 0.09 | 0.46 | - |
| MDS2 | - | - | -0.5 | -0.12 | - |
| MDS3 | - | - | -0.02 | -0.16 | - |
| Nauplii | - | - | - | - | -0.15 |
| Oithonoida | - | - | - | - | -0.16 |
| Pteropod | - | - | - | - | -0.13 |
| Salpidae | - | - | - | - | 0.27 |
| Total Abundance | - | - | 0.9 | 0.11 | -0.34 |
| Total Biomass | - | - | 1.2 | -0.02 | -0.36 |
| zoopk PC1 | - | - | 0.12 | -0.48 | - |
| zoopk PC2 | - | - | -0.49 | -0.01 | - |
