# Supplementary material for: Temporal changes in zooplankton indicators highlight a bottom-up process in the Bay of Marseille (NW Mediterranean Sea)
Source: PLoS One. 2023 Oct 23;18(10):e0292536. doi: 10.1371/journal.pone.0292536 (PMC10593231; doi:10.1371/journal.pone.0292536)
Supplement: S4 File — (DOCX) [file pone.0292536.s004.docx]

# **Supplementary information on zooplankton seasonality assessment.**

To assess zooplankton two seasonal sinusoidal models were performed for each variable:

$$Y=a_{1}.\sin\left( 2\pi.t/P \right)+a_{2}\cos\left( 2\pi.t/P \right)+b$$

Where Y is the monthly time series, a_1_ and a_2_ are the half amplitude of the sines and cosines sigmoid; t is the time, P is the period; and b, the intercept, is the average. For each series, two models were performed, one with one cycle per year (*P*=12) and one with two cycles per year (*P*=6). Akaike Information Criteria (AIC) was used to select which of the two models was the most likelihood model. Series were considered as seasonals when p-value < 0.05.

Table 1: Summary of the statistics (R^2^ of the sinusoidal model for zooplankton seasonality assessment. In bold significant seasonal patterns (p-value <0.05).

| Variable | One cycle (P=12) | | | Two cycles (P=6) | | |
| --- | --- | --- | --- | --- | --- | --- |
|  | R^2^ | pvalue | AIC | R^2^ | pvalue | AIC |
| **Biom 1000-2000** | 0.03 | 0.042 | -171 | **0.09** | **<0.001** | **-182** |
| **Biom 500-1000** | **0.12** | **<0.001** | **-120** | 0.11 | <0.001 | -117 |
| **Biom 300-500** | **0.18** | **<0.001** | **-136** | 0.11 | <0.001 | -121 |
| **Biom 200-300** | **0.12** | **<0.001** | **-159** | 0.03 | 0.083 | -140 |
| **Total Biomass** | **0.18** | **<0.001** | **-58** | 0.11 | <0.001 | -42 |
| **Calanoids** | **0.2** | **<0.001** | **105** | 0.1 | <0.001 | 127 |
| **Oithonoids** | **0.27** | **<0.001** | **81** | 0.04 | 0.034 | 136 |
| **Harpacticoids** | **0.03** | **0.036** | **70** | 0.03 | 0.077 | 71 |
| **Ergasilida** | **0.09** | **<0.001** | **-48** | 0.02 | 0.092 | -35 |
| **Nauplii** | **0.11** | **<0.001** | **21** | 0.05 | 0.01 | 33 |
| **Appendicularians** | **0.07** | **0.001** | **111** | 0.02 | 0.096 | 119 |
| **Cnidarians** | **0.17** | **<0.001** | **95** | 0.01 | 0.4 | 129 |
| **Chaetognaths** | **0.27** | **<0.001** | **80** | 0 | 0.624 | 139 |
| **Salps** | **0.09** | **<0.001** | **166** | 0.04 | 0.033 | 177 |
| **Crustaceans** | **0.47** | **<0.001** | **76** | 0 | 0.881 | 199 |
| **Fish eggs** | 0.05 | 0.006 | 145 | **0.06** | **0.004** | **144** |
| **Bivalves** | **0.09** | **<0.001** | **71** | 0.02 | 0.094 | 84 |
| **Pteropods** | **0.18** | **<0.001** | **78** | 0.01 | 0.258 | 113 |
| **Total abundance** | **0.25** | **<0.001** | **45** | 0.07 | 0.001 | 85 |
| **Zoopk PC1** | **0.17** | **<0.001** | **-1509** | 0.06 | 0.003 | -1484 |
| Zoopk PC2 | 0.02 | 0.23 | -1631 | 0.03 | 0.072 | -1633 |
| **MDS1** | **0.25** | **<0.001** | **-238** | 0.08 | <0.001 | -198 |
| **MDS2** | **0.05** | **0.011** | **-368** | 0.03 | 0.039 | -366 |
| **MDS3** | **0.31** | **<0.001** | **-456** | 0.05 | 0.01 | -396 |


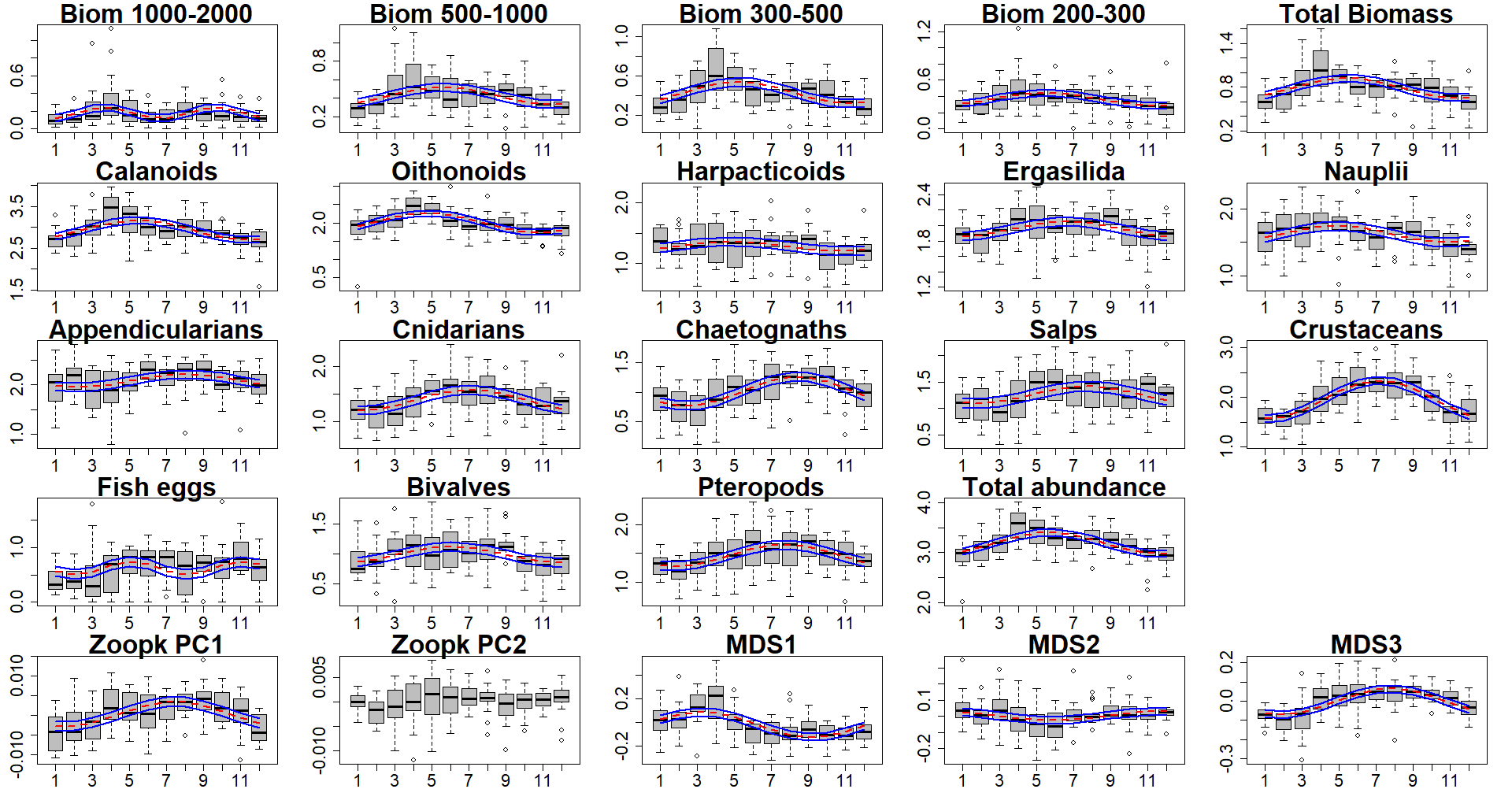


Figure 3: Boxplot of the seasonal (monthly) pattern of the zooplankton time series. When a model (with one or two cycle) was significant, the average prediction was represented by means of the red dashed line. The 95% confidence interval is represented by means of the blue lines. Series of size-fractions biomasses, in mg.m^-3^, (Biom 1000-2000, Biom 500-1000, Biom 300-500, Biom 200-300 µm, Total Biomass), *taxa* abundances and total zooplankton abundance, in individual.m^-3^, are log transformed (log10(x+1)).
